# Knockdown of Porf-2 restores visual function after optic nerve crush injury

**DOI:** 10.1038/s41419-023-06087-2

**Published:** 2023-08-28

**Authors:** Di Chen, Yi-Yu Sun, Lai-Yang Zhou, Xu Han, Shuo Yang, Fei-Yang Hong, Yuan Yuan, Xiao-Hua Wu, Guo-Hui Huang, Yuan-Chi Cheng, Ju Huang, Dong-Fu Feng

**Affiliations:** 1 Southern Medical University Affiliated Fengxian Hospital, Shanghai, 201499 China; 2 Department of Neurosurgery, Shanghai Ninth People’s Hospital, Shanghai Jiao Tong University School of Medicine, Shanghai, 201999 China; 3 State Key Laboratory of Medical Neurobiology and MOE Frontiers Center for Brain Science, Institutes of Brain Science, Fudan University, Shanghai, 200030 China; 4 Department of Critical Care Medicine, School of Anesthesiology, Naval Medical University, Shanghai, 200433 China; 5 Shanghai Jiao Tong University School of Medicine, Shanghai, 200025 China

**Keywords:** Trauma, Retina

## Abstract

Retinal ganglion cells (RGCs), the sole output neurons in the eyes, are vulnerable to diverse insults in many pathological conditions, which can lead to permanent vision dysfunction. However, the molecular and cellular mechanisms that contribute to protecting RGCs and their axons from injuries are not completely known. Here, we identify that Porf-2, a member of the Rho GTPase activating protein gene group, is upregulated in RGCs after optic nerve crush. Knockdown of Porf-2 protects RGCs from apoptosis and promotes long-distance optic nerve regeneration after crush injury in both young and aged mice in vivo. In vitro, we find that inhibition of Porf-2 induces axon growth and growth cone formation in retinal explants. Inhibition of Porf-2 provides long-term and post-injury protection to RGCs and eventually promotes the recovery of visual function after crush injury in mice. These findings reveal a neuroprotective impact of the inhibition of Porf-2 on RGC survival and axon regeneration after optic nerve injury, providing a potential therapeutic strategy for vision restoration in patients with traumatic optic neuropathy.

## Introduction

The axon bundle from the retinal ganglion cells (RGCs) forms the optic nerve and transmits signals from the eyes to the brain to generate vision [1, 2]. The somas and axons of RGCs are particularly vulnerable to injury in many pathological conditions, leading to permanent vision impairment [3, 4]. For patients with traumatic optic nerve injury, promoting RGC survival and axon regeneration is critical for the regeneration of retina-brain connections [1, 5]. Substantial progress has been made in the identification of the neuroprotective strategies to promote the survival rate and the length of regenerated axons of RGCs after injury [6–9]. However, the evidence supporting the recovery of visual function after optic nerve injury by a series of neuroprotective approaches is still very limited [1, 10, 11].

Preoptic regulatory factor-2 (Porf-2), also known as Cross GTPase activating protein (CrossGAP)/Vilse/ARHGAP39, was first identified in the 1990s in the hypothalamus in castrated male rats [12, 13]. Previous studies have revealed multiple biological functions of Porf-2, involving in the regulation of stem cell fate, cell migration, apoptosis, and proliferation [12, 14–16]. Porf-2 contributes to the process of neurodevelopment, as well as the learning and memory throughout the lifetime [17, 18]. Particularly, Porf-2 is involved in Roundabout (Robo)-mediated midline axon guidance in Drosophila and plays an important role in dendritic spine formation and synaptic plasticity [19, 20]. Our previous study shows that knockdown of Porf-2 accelerated axon growth and growth cone formation in hippocampal neurons by activating Rac1 through the GAP domain [21]. Thus, we want to further investigate whether Porf-2 regulates neuronal survival and axon regeneration after optic nerve injury.

In this study, we explored the role of Porf-2 and relevant signaling pathways in the regulation of the survival and axon regeneration of RGCs using the classical mouse optic nerve crush (ONC) injury model. We tested whether the manipulation of Porf-2 in the retina contributes to the preservation of functional vision in vivo. We found that knockdown of Porf-2 substantially protected RGCs from apoptosis and promoted long-distance optic nerve regeneration after ONC injury in both young and aged mice. Mechanistically, we found that Rac1 was a key downstream effector of Porf-2 in the regulation of RGC survival and axon regeneration after ONC injury. Notably, the Porf-2 knockdown-mediated protection of RGCs generates the preservation of functional vision in the entire visual pathway, as evidenced by the significant prevention of RGC complex degeneration, improved RGC function, and increased pupil constriction. Our study provides in-vivo supporting evidence that Porf-2 is a key regulator of RGC survival and axon regeneration after ONC injury, with an implication of therapeutic effect for vision preservation in patients with traumatic optic neuropathy.

## Results

### Inhibiting Porf-2 promotes RGCs survival and axon regeneration after ONC injury

We first investigated whether Porf-2 is expressed in RGCs. Using RNA-Scope and immunofluorescence, we found that Porf-2 co-localized with RBPMS, a marker of RGCs, suggesting that Porf-2 expresses in RGCs (Fig. S1A, B). Next, we examined whether the expression of Porf-2 was altered in RGCs after ONC injury by RT-qPCR, RNA-Scope and immunofluorescence (Fig. S1). The RT-qPCR’s result showed that Porf-2 was significantly upregulated 3 days after ONC injury (Fig. S1C). Compared to the intact RGCs, about 0.4 -fold increase of Porf-2 fluorescence intensity was observed in RGCs after ONC injury (Fig. S1D, E), suggesting that the expression of Porf-2 in RGCs is upregulated after ONC injury.

To explore whether inhibiting endogenous Porf-2 alters the injury response of RGCs, we employed the type2 adeno-associated virus (AAV2) carrying double Porf-2-targeting short hairpin RNAs (shRNA1-Porf-2 and shRNA2-Porf-2) to knock down the expression of endogenous Porf-2 in RGCs. The AAV containing scrambled hairpin RNA was used as the control (AAV2-shCtrl). We injected the combination of AAV2-shRNA1-Porf-2 and AAV2-shRNA2-Porf-2 (referred to as AAV2-shPorf-2), or AAV2-shCtrl as the control, into the vitreous body of 4-week-old C57BL/6 mice. We first confirmed that the AAV2 could transduce into the RGCs with a high efficiency of 91.00 ± 0.51% (Fig. S2A, C), which was consistent with our previous study [2]. The immunofluorescence’s results showed that compared to the AAV2-shCtrl, AAV2-shPorf-2 decreased the expression of Porf-2 in RGCs by 34.87% and 45.67%, respectively (Fig. S2B, D). The results of RT-qPCR also revealed that the expression level of Porf-2 was reduced two weeks after injection of AAV2-shPorf-2 (Fig. S2E).

**Fig. 1:**
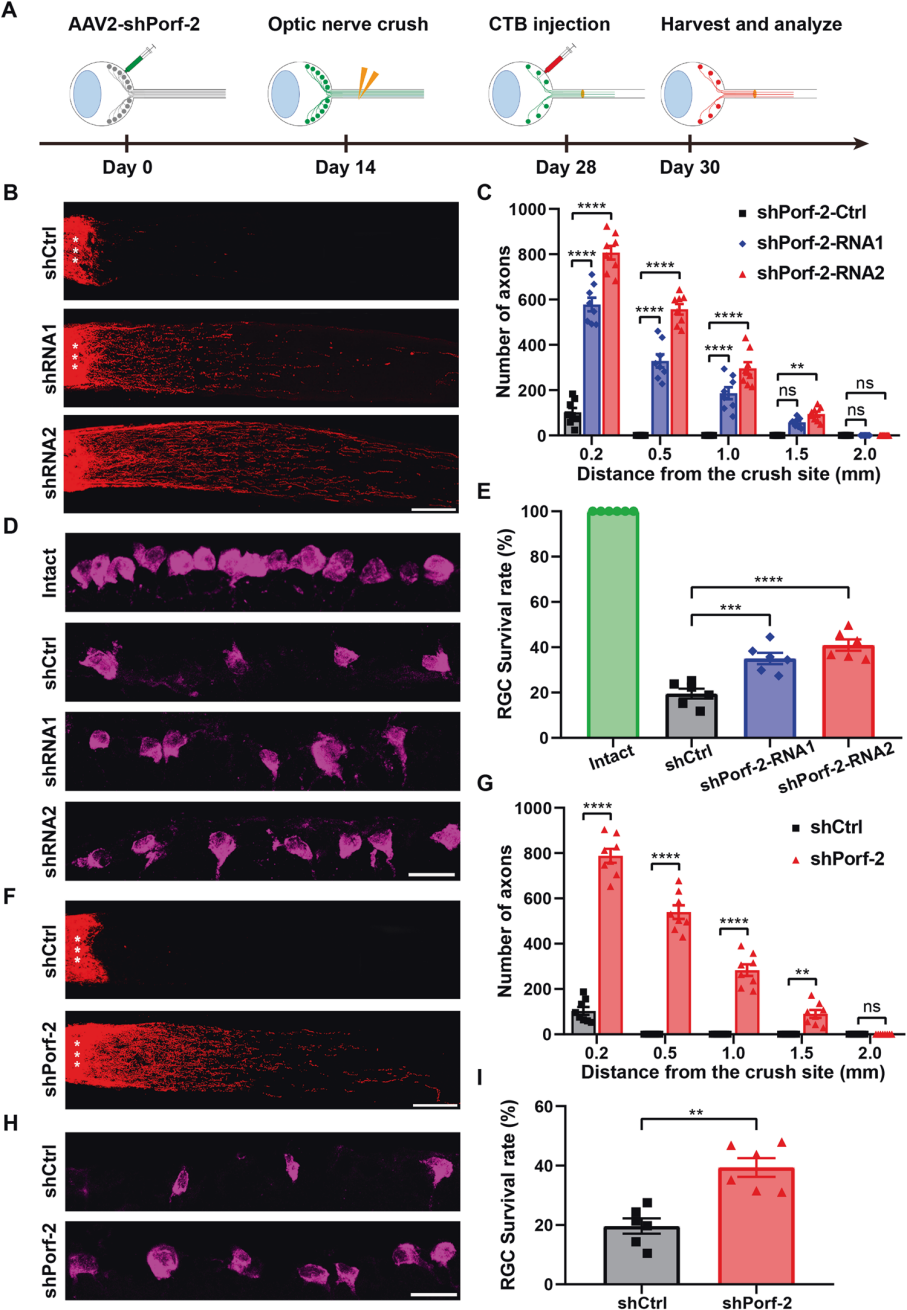
Knockdown of Porf-2 promotes RGC survival and axon regeneration after ONC injury. **A** Diagram of the experimental design. **B** Confocal images of optic nerves in young mice injected with AAV2-shPorf-2 (shPorf-2-RNA1 and shPorf-2-RNA2) or AAV2-shCtrl two weeks after ONC injury. Asterisks indicate the optic nerve crush site. Scale bar, 200 μm. **C** Quantification of optic nerve regeneration in (**B**) (two-way ANOVA followed by Bonferroni’s multiple-comparisons test, *p* < 0.0001 at 0.2, 0.50, and 1.00 mm from the crush site; *n* = 8 mice in each group). **D** Representative confocal images of retinal sections showing RBPMS^+^ RGCs (magenta) in young Porf-2-knockdown and control mice two weeks after ONC injury. Scale bar, 20 μm. **E** Quantification of the RGC survival rate in (**D**) (Mann–Whitney test, *n* = 6 mice in each group, at least eight non-adjacent retinal sections were analyzed for each mouse). **F** Confocal images of optic nerves in 12-month-old mice injected with AAV2-shPorf-2 (shPorf-2-RNA2) or AAV2-shCtrl two weeks after ONC injury. Asterisks indicate the optic nerve crush site. Scale bar, 200 μm. **G** Quantification of optic nerve regeneration in (**F**) (two-way ANOVA followed by Bonferroni’s multiple-comparisons test, *p* < 0.0001 at 0.2, 0.50, and 1.00 mm from the crush site; *p* < 0.01 at 1.5 mm from the crush site; *n* = 8 mice in each group). **H** Representative confocal images of retinal sections showing RBPMS^+^ RGCs (magenta) in 12-month-old Porf-2-knockdown (shPorf-2-RNA2) and control mice two weeks after ONC injury. Scale bar, 20 μm. **I** Quantification of the RGC survival rate in (**H**) (Mann–Whitney test, *n* = 6 mice in each group, at least eight non-adjacent retinal sections were analyzed for each mouse). Data are presented as the mean ± SEM. **p < 0.01,***p < 0.001, ****p < 0.0001. ns, not significant.

We performed the optic nerve crush injury two weeks after the intravitreal injection of AAV2-shPorf-2 (or AAV2-shCtrl as the control) in 4-week-old C57BL/6 mice. The optic nerve regeneration was assessed two weeks after the optic nerve crush. The regenerating axons were labeled by the anterogradely tracer cholera toxin subunit B (CTB) which was injected into the vitreous cavity two days prior to tissue harvest (Fig. 1A). To assess the effects of injected and non-injected shCtrl on axonal regeneration and RGC survival after ONC, we conducted experiments and the results indicated that both the injected and non-injected shCtrl did not have a significant impact on axonal regeneration and RGC survival (Fig. S3A–D). Then, in contrast to the controls where very few injured axons sprouted across the injury site, shRNAs-mediated knockdown of Porf-2 resulted in a significant increase in axon regeneration (Fig. 1B). We found that inhibition of Porf-2 significantly promoted the regenerated axons in both the number and the length, with some axons growing up to 2.0 mm by two weeks post-ONC injury (Fig. 1C). Next, we assessed the RGC survival rate by immunostaining with anti-RBPMS antibody to label RGCs in the retinal sections. As shown in Fig. 1D and E, in contrast to approximately 20% survival in the control group, the survival rates of RGCs in mice treated with AAV2-shRNA1-Porf-2 or AAV2-shRNA2-Porf-2 were 35.05% and 40.97%, respectively. Furthermore, we evaluated the RGC survival rate by immunostaining with anti-beta III tubulin (Tuj1) antibody to label RGCs in the whole-mount retinas, and we observed a similar increased survival rate (Fig. S4A, C). These findings suggest that knockdown of Porf-2 promotes axon regeneration and RGCs survival rate upon ONC injury in young mice.

Many regenerative approaches that work well in young individuals often fail in older individuals [4, 22, 23]. To investigate whether inhibition of Porf-2 promoted RGCs survival in aged mice, we performed the above experiments in 12-month-old C57BL/6 mice. We conducted an ONC injury in the old adult mice two weeks after the injection of AAV2-shPorf-2 (or AAV2-shCtrl as the control). Similar to what was observed in young mice, we found that shRNA-mediated knockdown of Porf-2 significantly promoted axon regeneration after ONC injury in 12-month-old mice (Fig. 1F, G). Similar to the result observed in young mice, knockdown of Porf-2 doubled the survival rate of RGCs in old adult mice after ONC injury, compared to the control group (Fig. 1H, I). These data suggest that aging does not diminish the beneficial effects of Porf-2 knockdown on axon regeneration and RGC survival. Taken together, these findings clearly indicate that knockdown of Porf-2 is sufficient to induce significant optic nerve regeneration after ONC injury and provide a critical protective effect on RGC survival in both young and aged mice.

### Knockdown of Porf-2 induces axon initiation and long-term axon regeneration after ONC injury

**Fig. 2:**
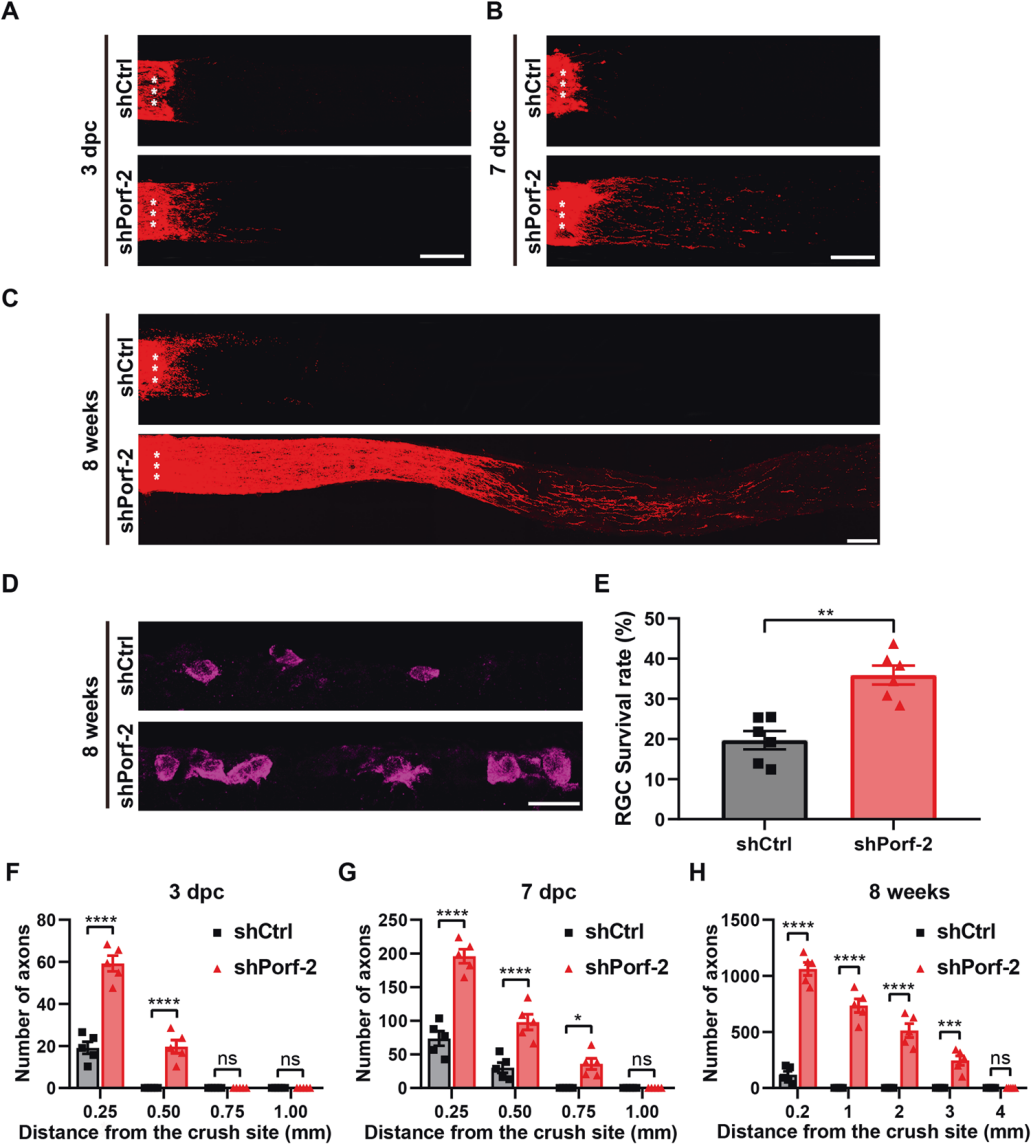
Knockdown of Porf-2 induces axon initiation and long-term axon regeneration after ONC injury. **A–C** Representative confocal images of optic nerves in Porf-2-knockdown and control RGCs at 3 days, 7 days and 8 weeks post-ONC injury. Asterisks indicate the optic nerve crush site. Scale bar, 200 μm. **D** Representative confocal images of retinal sections showing RBPMS^+^ RGCs (magenta) from Porf-2 knockdown and control mice eight weeks after ONC injury. Scale bar, 20 μm. **E** Quantification of the RGC survival rate in **D** (Mann–Whitney test, *p* < 0.01; *n* = 6 mice in each group, at least eight non-adjacent retinal sections were analyzed for each retina). **F**–**H** Quantification of optic nerve regeneration at 3 days, 7 days and 8 weeks post-ONC (two-way ANOVA followed by Bonferroni’s multiple-comparisons test, *n* = 5 mice in each group). Data are presented as the mean±SEM. *p < 0.05, **p < 0.01, ***p < 0.001,****p < 0.0001. ns, not significant.

To examine whether Porf-2 affects the initiation and elongation of axon regeneration, we analyzed axon regeneration at shorter time points after injury in Porf-2 knockdown mice. On the 3rd day post-ONC injury (3 dpc), we observed that the CTB-labeled axons stopped at the crush site in the control mice injected with AAV2-shCtrl, with no labeled fibers found distal to the lesion site. Conversely, in the Porf-2 knockdown mice, a substantial amount of short sprouts could be observed across the lesion site, suggesting that knockdown of Porf-2 stimulates the initiation of axon regeneration (Fig. 2A, F). On the 7th day post-ONC injury (7dpc), this phenotype was further intensified, and more regenerating axons were observed beyond the crush site in Prof-2 knockdown mice (Fig. 2B, G). Together, these results demonstrate that knockdown of Porf-2 promotes both the initiation and elongation of axon regeneration after ONC injury.

To further determine whether knockdown of Porf-2 influences long-term axon regeneration, we assessed the optic nerve regeneration 8 weeks after ONC injury and found that knockdown of Porf-2 significantly increased both the number and length of regenerating axons. Remarkably, most optic nerves had regenerating axons extending 2 mm from the crush site, with some axons reaching the optic chiasm (Figs. 2C, H and S4B). Additionally, we performed an evaluation of RGC survival at eight weeks after ONC. The results demonstrated a significantly higher survival rate of RGCs with knockdown of Porf-2 compared to the control group at this time point (Fig. 2D, E). Collectively, these results demonstrate that knockdown of Porf-2 promotes long-term axon regeneration and RGCs survival after ONC injury.

### Knockdown of Porf-2 accelerates axon growth and growth cone formation in retinal explants

**Fig. 3:**
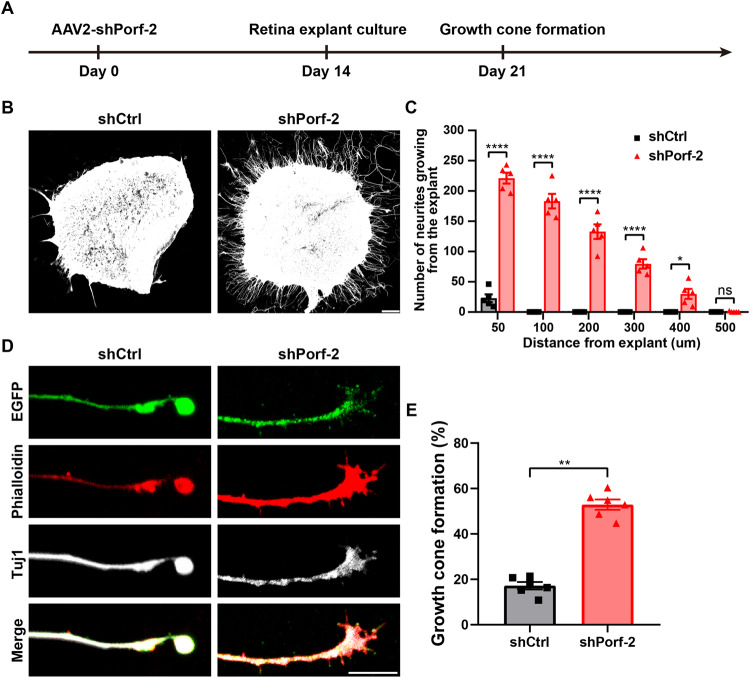
Knockdown of Porf-2 accelerates axon growth and growth cone formation in retinal explants. **A** Diagram of the experimental design. **B** Representative microphotographs of retinal explants stained with anti-beta III tubulin (Tuj1) showing neurite growth in AAV2-shCtrl and AAV2-shPorf-2 groups. Scale bar, 100 μm. **C** Quantification of neurites counted at different distances from the edges of the explants in (**B**) (two-way ANOVA followed by Bonferroni’s multiple-comparisons test, *p* < 0.0001 at 50, 100, 200, and 300 μm from the edges of the explants; *p* < 0.05 at 400 μm from the edges of the explants; *n* = 5 mice in each group). **D** Representative confocal images of neurites at the end of an axon in retinal explants from AAV2-shCtrl and AAV2-shPorf-2 groups stained with AAV2-EGFP (green), phalloidin (red), and Tuj1 (gray). Scale bar, 5 μm. **E** Percentage of growth cone-forming neurites in retinal explants in **D** (Mann–Whitney test, *p* < 0.01; *n* = 6 mice in each group, six to eight growth cones were analyzed for each retinal explant). Data are presented as the mean ± SEM. *p < 0.05, **p < 0.01, ****p < 0.0001. ns, not significant.

Because the formation of axon growth cones is a key step in the initiation of axonal regeneration [24–26], we next evaluated whether Porf-2 is involved in this process. We employed an ex vivo explant culture system. Two weeks after the intravitreal injection of AAV2-shRNA2-Porf-2 (or AAV2-shCtrl as the control) in 4-week-old mice, we dissected the retinas and placed them in culture (Fig. 3A). One week later, we harvested the retinal tissues in culture and performed immunostaining with an anti-Tuj1 antibody. We found that a large number of axons grew from the edges of the explants from the mice injected with AAV2-shPorf-2, with some axons growing up to 300 μm. In contrast, very few axons sprouted across the edge of the explants from the mice treated with AAV2-shCtrl (Fig. 3B, C)

In addition, we performed immunostaining using anti-Tuj1 and phalloidin to visualize the morphology of axons and growth cones in the retinal explants. In AAV2-shCtrl-treated mice, a significant percentage of axons had retraction bulbs at their ends. In AAV2-shPorf-2 treated mice, knockdown of Porf-2 significantly decreased the formation of retraction bulbs, and the growth cones were almost intact. These findings indicate that knockdown of Porf-2 effectively transformed dystrophic axon tips into growth cones, thereby facilitating axon regeneration (Fig. 3D, E). These results provide direct supporting evidence that knockdown of Porf-2 can significantly accelerate axon growth and growth cone formation in retinal explants.

### Porf-2 affects axon regeneration and growth cone formation by altering the activity of Rac1

Most molecules known to regulate optic nerve regeneration largely act through three molecular pathways: mTORC1, GSK3β and STAT3 pathway [6, 8, 27, 28]. To examine whether Porf-2 affects axon regeneration and growth cone formation through these pathways, we measured the levels of Ser235/236 phosphorylation of S6 ribosomal protein (pS6, an indicator of mTORC1 activity) and phosphor-GSK-3β serine 9 residue (pGSK3β, a marker of GSK3β activity using immunohistochemistry (Fig. S5A, B). Interestingly, we found that both the levels of pS6 and pGSK3β in RGCs were unchanged following the intravitreal injection of AAV2-shPorf-2 (Fig. S5D, E). To further validate the above results, we conducted a western blotting assay to assess the changes in pS6, p4EBP1 (another marker of mTORC1 activity), pGSK3β, and pSTAT3 following Porf-2 knockdown (Fig. S5C). The results indicated that the levels of pS6, p4EBP1, pGSK3β, and pSTAT3 in the retina remained unaltered after the inhibition of Porf-2 (Fig. S5F–I). These findings suggest that the knockdown of Porf-2 does not affect the mTORC1, GSK3β, and STAT3 pathways.

Our previous study reported that knockdown of Porf-2 in hippocampal neurons in vitro drastically promoted the formation of growth cones and axon growth by regulating the activity of Rac1. Therefore, we reasoned that Porf-2 might affect RGC survival and axon regeneration after ONC injury by altering Rac1 activity. Then, we assessed the alteration of Rac1 activity by Rac1-GTP/total Rac1 using the Rac1 Activation Assay Kit, and found that the relative activity of Rac1 has increased by 0.73-fold before ONC injury and by 0.75-fold at 3 days post-ONC injury in the retinas of the mice injected with AAV2-shPorf-2, compared to the controls (Fig. S6A, D). These results indicate that knockdown of Porf-2 enhances the activity of Rac1 in both intact and injured conditions.

**Fig. 4:**
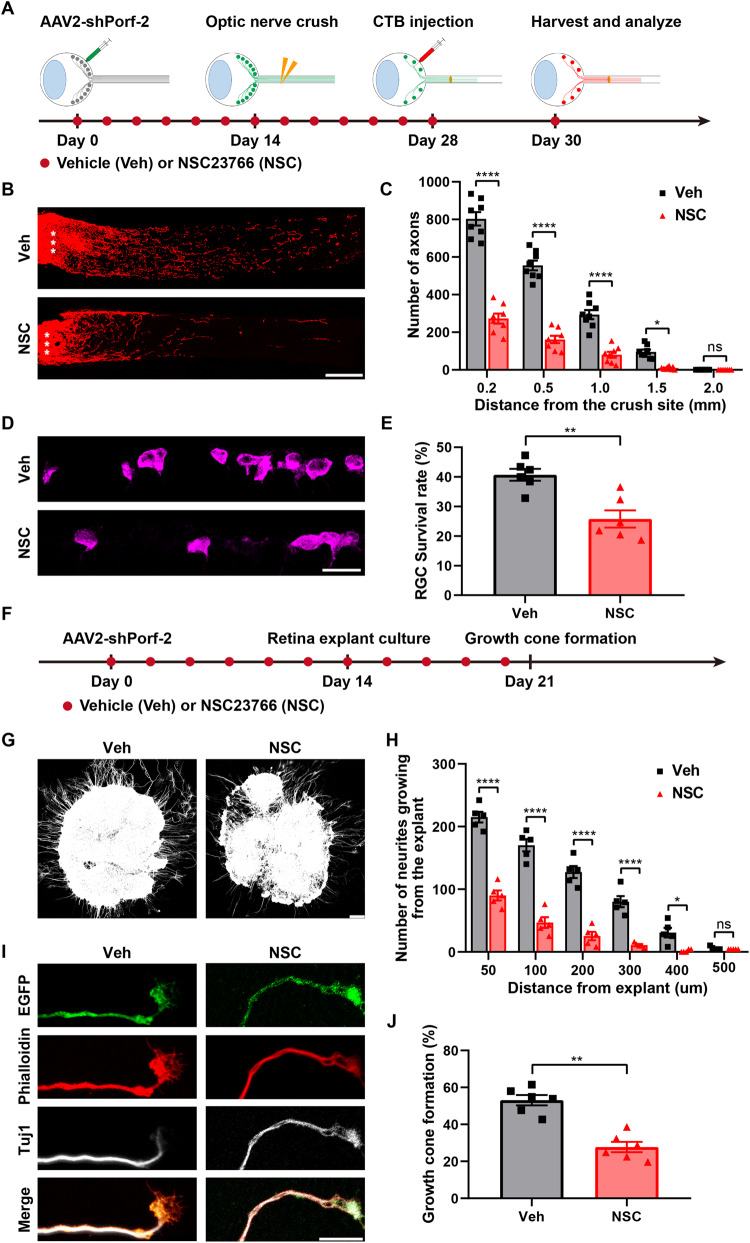
Porf-2 affects axon regeneration and growth cone formation by altering the activity of Rac1. **A** Experimental timeline. Vehicle (Veh) or NSC23766 (NSC) was administered intraperitoneally once every two days after AAV2-shPorf-2 injection. **B** Confocal images of optic nerves from mice injected with AAV2-shPorf-2 14 days before ONC injury and treated with either Veh or NSC intraperitoneally. Asterisks indicate the optic nerve crush site. Scale bar, 200 μm. **C** Quantification of optic nerve regeneration in **B** (two-way ANOVA followed by Bonferroni’s multiple-comparisons test, *p* < 0.0001 at 0.20, 0.50, and 1.00 mm from the crush site; *p* < 0.05 at 1.50 mm from the crush site; *n* = 8 mice in each group). **D** Representative confocal images of retinal sections showing surviving RBPMS^+^ RGCs in the NSC-injected and control mouse groups. Scale bar, 20 μm. **E** Quantification of the RGC survival rate in **D** (Mann–Whitney test, *p* < 0.01; *n* = 6 mice in each group, at least eight non-adjacent retinal sections per mouse). **F** Experimental timeline. For in vivo studies, Veh or NSC was administered intraperitoneally once every two days after the AAV2-shPorf-2 injection. For in vitro experiments, Veh or NSC was added to the retinal explant medium, with the medium being changed once every two days. **G** Representative microphotographs of retinal explants stained with anti-beta III tubulin (Tuj1) showing neurite growth in Veh- or NSC-treated explants. Scale bar, 100 μm. **H** Quantification of neurites at different distances from the edges of the explants in (**G**) (two-way ANOVA followed by Bonferroni’s multiple-comparisons test, *p* < 0.0001 at 50, 100, 200, and 300 μm from the edges of the explants; *p* < 0.05 at 400 μm from the edges of the explants; *n* = 5 mice in each group). **I** Representative confocal images of neurites at the end of an axon in retinal explants from Veh- or NSC-treated groups stained with AAV2-EGFP (green), phalloidin (red), and Tuj1 (gray). Scale bar, 5 μm. **J** Percentage of growth cone-forming neurites in retinal explants in **I** (Mann–Whitney test, *p* < 0.01; *n* = 6 mice in each group, six to eight growth cones were analyzed for each retinal explant). Data are presented as the mean ± SEM. *p < 0.05, **p < 0.01, ****p < 0.0001. ns, not significant.

To further examine the necessity of Rac1 activation for the impact of Porf-2 on the regulation of RGC survival and axon regeneration, we administered the NSC23766, a selective inhibitor of Rac1 [6, 29, 30], in the above experiment. First, we assessed the changes in Rac1 activity two weeks after injection of NSC23766. We found that NSC23766 decreased Rac1 activity in the retinas of the mice treated with AAV2-shCtrl or AAV2-shPorf-2, suggesting that NSC23766 can be used as a specific Rac1 inhibitor (Fig. S6B, C, E, F). We proceeded to administer NSC23766 and the vehicle control intraperitoneally every two days following AAV2-shPorf-2 injection (Fig. 4A). We found that the number and length of regenerating axons were significantly reduced in the mice injected with NSC23766 compared to vehicle-treated mice (Fig. 4B, C). We also evaluated RGC survival after NSC23766 treatment and found that the enhanced survival of RGCs induced by Porf-2 knockdown was largely blocked by NSC23766 (Fig. 4D). The survival rate of RGCs in NSC23766-treated mice decreased by approximately 15% compared to the vehicle-injected mice (Fig. 4E). Taken together, these results demonstrate that NSC23766 can block the effect of Porf-2 knockdown on the RGC survival and axon regeneration after ONC injury.

We also investigated the effect of Rac1 activation on Porf-2-mediated modulation of axon growth and growth cone formation in retinal explants. As shown in Fig. 4F, two weeks following the intravitreal injection of AAV2-shPorf-2, retinas were dissected and cultured in a culture medium containing NSC23766 (or the vehicle control) for 1 week. We observed that the treatment of NSC23766 significantly decreased the number and length of axons extending from the retinal explants transduced with AAV2-shPorf-2, compared to those treated with the vehicle (Fig. 4G, H). Furthermore, whereas the growth cone morphology was almost intact in the vehicle control, NSC23766 treatment resulted in a reduction in the growth cone area and a partially collapsed morphology (Fig. 4I, J). These results suggest that blocking Rac1 activation can prevent the effects of Porf-2 knockdown on axon growth and growth cone formation in retinal explants. Taken together, our results demonstrate that Rac1 is a key downstream effector of Porf-2. It is necessary for promoting RGC survival and axon regeneration after ONC injury, as well as for boosting axon growth and growth cone formation in retinal explants.

### Knockdown of Porf-2 facilitates the recovery of visual function after ONC injury

**Fig. 5:**
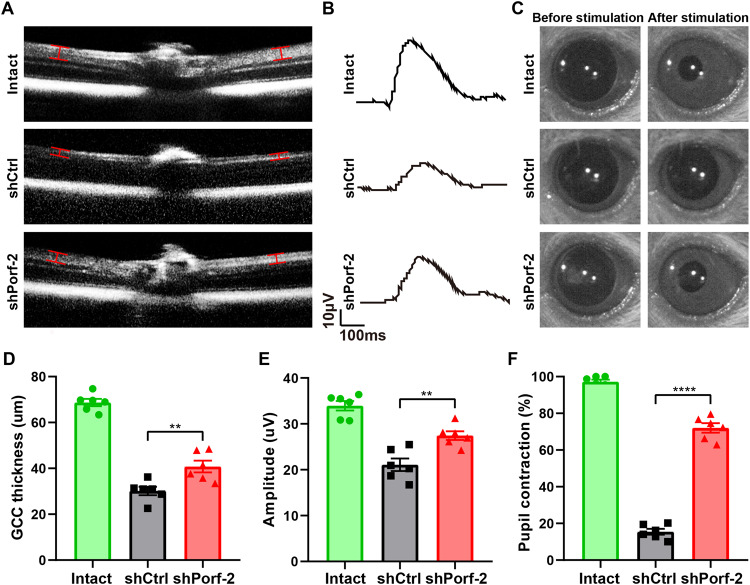
Knockdown of Porf-2 facilitates the recovery of visual function after ONC injury. **A** Representative individual B-scan images (scan 1 at 0° in en face images) from AAV2-shCtrl-injected and AAV2-shPorf-2-injected mice before ONC injury and 3 weeks after ONC injury. A vertical caliper was placed on each side of the optic nerve head, 500 µm away from the center of the optic nerve head. The caliper was used to determine the thickness of the ganglion cell complex (GCC), comprising the three innermost retinal layers: the nerve fiber layer (NFL), the ganglion cell layer (GCL), and the inner plexiform layer (IPL). Scale bar, 200 µm. **B** Representative pSTR amplitudes from mice injected with AAV2-shCtrl or AAV2-shPorf-2 before ONC injury and 3 weeks after ONC injury. The pSTR amplitudes were assessed at a flash intensity of 3 × 10^-5^cd.sm^-2^. The pSTR was measured from the baseline to the positive peak of the waveform. Scale bar, 10 μV, 100 ms. **C** Representative pupil changes from mice injected with AAV2-shCtrl or AAV2-shPorf-2 before ONC injury and 8 weeks after ONC injury. The contraction of the pupil is the area of the pupil before light exposure minus the area of the pupil after light exposure, divided by the area of the pupil before light exposure. **D** Quantification of the thickness of the GCC in (**A**) (Mann Whitney test, *p* < 0.01; n = 6 mice in each group). **E** Quantification of the pSTR amplitudes in (**B**) (Mann–Whitney test, *p* < 0.01; *n* = 6 mice in each group). **F** Quantification of the pupil changes in (**C**) (Mann–Whitney test, *p* < 0.0001; *n* = 6 mice in each group). Data are presented as the mean ± SEM. **p < 0.01, ****p < 0.0001.

To evaluate the effect of Porf-2 on retina morphology, we carried out spectral-domain optical coherence tomography (SD-OCT) scanning to measure the thickness of the retinal ganglion cell complex (GCC). The thickness of the GCC (including the nerve fiber layer [NFL], ganglion cell layer [GCL], and inner plexiform layer [IPL]) was 68.70 ± 1.58 μm in uninjured mice, but decreased to 30.14 ± 1.82 μm three weeks after ONC injury (Fig. 5A, D). Importantly, knockdown of Porf-2 significantly increased the GCC thickness to 40.78 ± 2.57 μm (Fig. 5A, D). These results provide supporting evidence that knockdown of Porf-2 prevents the retinal GCC from degeneration after ONC injury.

To further test whether the AAV2-shPorf-2-mediated protection of RGCs can preserve vision after injury, we investigated whether knockdown of Porf-2 maintains the RGC function by using the positive scotopic threshold response electroretinogram (pSTR-ERG), in which the RGC activity in response to different visual stimuli was measured. pSTR-ERG responses were readily detectable in uninjured retinas (33.96 ± 1.03 μV), but were significantly reduced at three weeks post-ONC injury (21.08 ± 1.36 μV), reflecting a severe loss of RGC functionality after injury (Fig. 5B, E). Significantly, knockdown of Porf-2 preserved pSTR-ERG responses to the levels similar to those recorded in the uninjured retina (27.44 ± 0.95 μV; Fig. 5B, E). These results indicate that knockdown of Porf-2 improves the function of RGCs after ONC injury.

Next, we assessed whether RGCs could transmit visual information to the brain in vivo. We performed pupillary light reflex (PLR) to measure the pupil constriction of mice from the uninjured, ONC injured, and Porf-2 knockdown groups. Pupil constriction was extremely prominent in the uninjured animals and was markedly reduced at eight weeks post-ONC injury (15.46 ± 1.61%; Fig. 5C, F). Remarkably, pupil constriction was significantly increased in Porf-2 knockdown mice (72.04 ± 2.59%). Our results demonstrate that knockdown of Porf-2 improves the functionality of the entire visual pathway from the retina to the brain, after ONC injury. Taken together, these results provide supporting in vivo evidence that knockdown of Porf-2 enhances RGC protection and promotes the recovery of visual function after ONC injury.

### Knockdown of Porf-2 after ONC injury facilitates RGC survival and axon regeneration

**Fig. 6:**
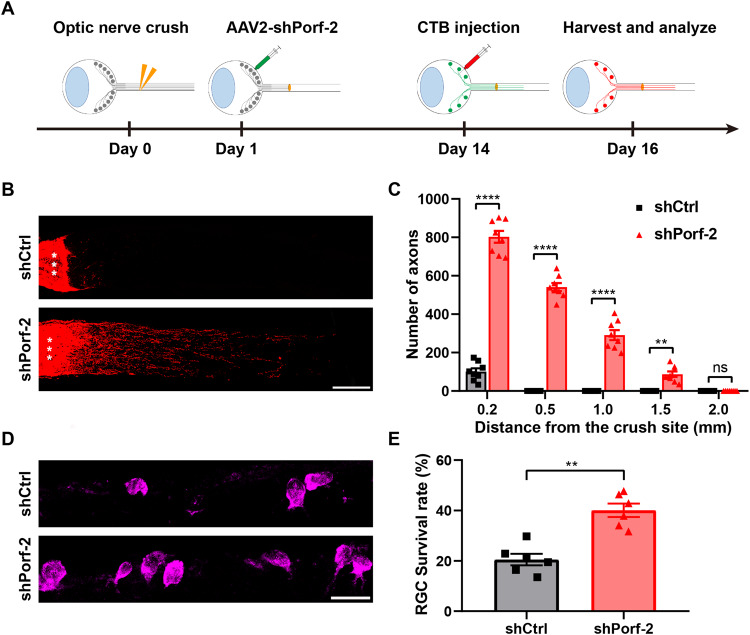
Knockdown of Porf-2 after ONC injury boosts RGC survival and axon regeneration. **A** Diagram of the experimental design. **B** Representative confocal images of optic nerves from Porf-2-knockdown and control RGCs two weeks after ONC injury. Asterisks indicate the optic nerve crush site. Scale bar, 200 μm. **C** Quantification of optic nerve regeneration in (**B**) (two-way ANOVA followed by Bonferroni’s multiple-comparisons test, *p* < 0.0001 at 0.2, 0.5, and 1.0 mm from the crush site; *p* < 0.01 at 1.5 mm from the crush site; *n* = 8 mice in each group). **D** Representative confocal images of retinal sections showing RBPMS^+^ RGCs (magenta) from Porf-2 knockdown and control mice two weeks after ONC injury. Scale bar, 20 μm. **E** Quantification of the RGC survival rate in (**D**) (Mann–Whitney test, *p* < 0.01; *n* = 6 mice in each group, at least eight non-adjacent retinal sections were analyzed for each retina). Data are presented as the mean ± SEM. **p < 0.01, ****p < 0.0001. ns, not significant.

To investigate the translational potential of AAV2-shPorf-2, we tested if post-injury knockdown of Porf-2 could also promote RGC survival and axon regeneration. We performed ONC injury on 4-week-old mice and injected AAV2-shPorf-2 or AAV2-shCtrl into the vitreous body of these mice 1 day after ONC injury. Optic nerve regeneration was assessed two weeks after ONC injury (Fig. 6A). As expected, few injured axons sprouted across the crush site in the mice treated with AAV2-shCtrl. In contrast, a large number of regenerating axons were observed in the mice treated with AAV2-shPorf-2, with some axons reaching 1.5 mm from the crush site (Fig. 6B, C). Next, we assessed RGC survival by anti-RBPMS immunostaining in the retinal sections. As shown in Fig. 6D and E, the survival rate of RGCs in mice treated with AAV2-shPorf-2 was approximately 40%, compared to the approximately 20% survival in the control group. These results clearly demonstrate that post-injury treatment with AAV2-shPorf-2 effectively facilitates axon regeneration and promotes RGC survival, indicating that inhibition of Porf-2 may potentially be applied to treat diseases involving optic nerve damage.

## Discussion

Damaged neurons in the adult mammalian central nervous system (CNS) often have a limited capacity to regenerate axons and form new synapses, and in many cases, injured neurons die, which ultimately leads to permanent functional deficits [1, 3, 31]. Whether the manipulation of a critical regulator of neuron survival can protect neurons from injury and preserve their function is an area of active research [1, 2]. Here, we identified a novel role for Porf-2 in regulating the survival and regeneration of RGCs following ONC injury using the classical mouse optic nerve crush model. We found that knockdown of Porf-2 by AAV2 infection largely improved the survival rate of RGCs, induced regeneration of the optic nerve, and dramatically promoted the recovery of visual function after ONC injury in mice. Mechanistically, we found that Porf-2 exerts its function on RGCs by regulating the activity of Rac1; the regenerative effect of Porf-2 knockdown could be blocked by treatment with the Rac1 inhibitor, NSC23766. Thus, our results indicate that the inhibition of Porf-2 via AAV2 gene therapy may be a very promising strategy for treating traumatic optic neuropathy.

Porf-2, a member of the Rho GAP group, has been primarily studied in the context of CNS development, especially axon outgrowth and guidance and dendritic spine or synapse formation [12, 32]. Previous research by our team revealed that the inhibition of Porf-2 could promote the formation of growth cones and axons in hippocampal neurons [21]; however, the function and molecular mechanisms of Porf-2 in neuron survival and axon regeneration after injury in mammals remained unclear. To address this problem, we packaged two AAV2 viruses (AAV2-shRNA1 and AAV2-shRNA2) for the knockdown of Porf-2 and discovered that knocking down Porf-2 significantly enhanced axon regeneration and RGC survival two weeks after ONC injury. These findings were very exciting as they highlight the extremely promising potential of Porf-2 knockdown as a means for preserving visual function following injury.

Ample evidence indicates that the regenerative capacity of axons after injury often declines with age [4, 33]. Previous studies have revealed that genetic deletion of the mTOR inhibitor, PTEN, in young animals prevents axotomy-induced reduction of mTOR activity and promotes the regeneration of both RGCs and corticospinal tract axons after CNS injury, whereas axon regeneration distal to injury is greatly diminished in aged mice (up to 18 months old) [6, 22, 34]. In our study, the capacity for axonal regeneration and RGC survival after ONC injury remained unchanged even in 12-month-old mice following knockdown of Porf-2, suggesting that aging did not greatly reduce the beneficial effect of Porf-2 knockdown on these processes. In addition, recent studies have reported that the knockdown or overexpression of some genes can promote axonal regeneration and the survival of RGCs after ONC injury, but these effects are usually transient, and most RGCs continue to die after a few weeks [11, 24]. Here, we observed that knockdown of Porf-2 significantly increased the lengths and number of regenerating axons even 8 weeks after ONC injury, with some axons reaching the optic chiasm. Thus, knockdown of Porf-2 promotes sustained and long-term axon regeneration after ONC injury.

Importantly, we also determined the mechanism by which Porf-2 affects the survival and regeneration of RGCs after ONC injury. The mTOR and GSK3β signaling pathways, which function in the soma to support the intrinsic capacity for axon regeneration, have well-known roles in RGC biology [24]. Interestingly, knockdown of Porf-2 had no effect on these two pathways, suggesting that the expression of genes regulating intrinsic axon regeneration pathways may be unaltered. Notably, Hu et al. found that Porf-2 can directly regulate the midline crossing of axons via Rac1-dependent cytoskeletal alterations, which play a critical role in axon development in the CNS [20]. Lim et al. also showed that Porf-2 is involved in dendritic spine formation and synaptic plasticity via the Rac1 pathway [18]. Recently, our team revealed that Porf-2 negatively regulates axon extension and growth and inhibits growth cone dynamics by altering Rac1 activity [21]. Indeed, many studies have suggested that selective activation of Rac1 could improve the survival and regeneration of RGCs after injury, and Rac1 activation is a therapeutic strategy for counteracting neuronal degeneration [35–38]. In line with these previous reports, we discovered that AAV2-shPorf-2 injection greatly increased Rac1 activity. Additionally, in both in vivo experiments in mice and in vitro experiments using retinal explants, treatment with the Rac1 inhibitor, NSC23766, dramatically neutralized the pro-regeneration effects of Porf-2 knockdown on RGCs, highlighting the necessity of Rac1 activation for achieving the regenerative phenotype. Although the residual axons observed in NSC23766-treated mice or retinal explants may reflect the involvement of other pathways downstream of Porf-2, these data suggest that Rac1 activation plays a major role in facilitating the effects of Porf-2 knockdown.

Notably, few studies have reported the recovery of visual function after optic nerve injury [1, 31]. Intriguingly, inhibition of Porf-2 promoted the recovery of visual function, as evidenced by the prevention of retinal GCC degeneration, significantly improved RCG function, and dramatically increased pupil constriction. Furthermore, knockdown of Porf-2 in RGCs after ONC injury promoted significant optic nerve regeneration, highlighting the promise of Porf-2 inhibition as a therapeutic strategy for the treatment of nerve injury.

In summary, our results firmly support the notion that Porf-2 is a pivotal regulator of axon regeneration and RGC survival after ONC injury. Given that AAV vectors have good safety profiles and are frequently used to treat neurological conditions, we propose AAV-mediated Porf-2 inhibition as a general therapeutic target for promoting RGC protection and vision preservation following optic nerve injury or disease [2, 39, 40]. The manipulation of the Porf-2 gene via AAV vectors may be a promising and valuable approach for rescuing retinal ganglion cells and treating traumatic optic neuropathy.

## Materials and methods

### Animals

C57BL/6 mice were purchased from SLAC Laboratory Animal (Shanghai, China). All experimental protocols were approved by the Institutional Animal Care and Use Committee at Shanghai Jiaotong University School of Medicine (Protocol A-2019-060). Mice were housed under controlled temperatures (25 °C) in a 12-hour light/dark cycle (light time, 7 AM to 7 PM) with water and ad libitum chow.

### AAV plasmid construction and preparation

The Institutional Biosafety Committee of the Shanghai Jiao Tong University School of Medicine approved AAV procedures. AAV serotype 2/2 was used for all AAVs. The AAV construct backbone (AAV2-CAG-EGFP-WPRE-SV40pA) for shRNAs was purchased from Taitool Bioscience Co. (Shanghai, China). Two targeting sequences against Porf2 (shRNA1, GCCCTTGATTCCTCATGAA; shRNA2, GCACGTAGCCCTAGAGATA) were cloned into pAAV plasmids. The AAV titer used for this study was 1.02E + 13 v.g./mL.

### Intravitreal injection and optic nerve crush injury

Intravitreal virus injection, ONC injury, axon labeling, and RGC survival analyses were performed as described previously [2, 6, 24]. Briefly, under anesthesia, 2 μL AAV2-shRNA was injected into the left vitreous humor of a mouse using a WPI Nanofil syringe (35-gauge needle). Two weeks later, the left optic nerve was exposed intraorbital and crushed approximately 1 mm behind the optic disc using a pair of Dumont #5 forceps (Fine Science Tools) for 5 s. To label RGC axons in the optic nerve, 1.5 μL Alexa Fluor 555-conjugated CTB (2 mg/ml, Thermo Fisher Scientific) was injected into the left vitreous humor using a WPI Nanofil syringe (35-gauge needle) two days before the mouse was sacrificed by transcardial perfusion under anesthesia. For the post-injury treatment model, all steps were performed in the same way except that intravitreal viral injection was performed one day after the ONC injury.

### Retina explant culture

Retinal explant dissection and culturing were performed as described previously [41, 42]. Two weeks after intravitreal injection of AAV2-shPorf-2 or AAV2-shCtrl, mouse eyes were quickly removed using Dumont #5 forceps (Fine Science Tools) and placed into ice-cold Neurobasal-A medium (Invitrogen, 10888022) without calcium and magnesium. Under the anatomic microscope, the dissected eyes were cut open to obtain the retina, which was cut into small pieces approximately 750 μm in diameter with a punch needle. Then, the Neurobasal-A medium was removed from coverslips previously coated with poly-L-lysine and laminin, and the retina explants with an RGC layer were placed face down on the coated side of the glass coverslip. Finally, retinal explants were cultured in Neurobasal-A medium with the following supplements: 2% B27 (Invitrogen, 0080085-SA), 0.5% L-Glutamine (Corning, 25-005-CI), 0.4% methylcellulose (Sigma-Aldrich, M0512), 100 units/mL penicillin–100 μg/mL streptomycin (Invitrogen, 15140122). Note that all explants adhered to the glass coverslip. If an explant became detached, it was removed from the culture. Retinal explants were cultured at 37 °C with 5% carbon dioxide for one week.

### Histology and immunostaining of retinal sections, whole-mount retinas, and retinal explants

Histology and immunostaining were conducted as described in our previous study [2]. Briefly, eyes with the attached optic nerve segment, which had been surgically removed from perfused mice, were post-fixed in 4% PFA for two hours. Typically, 20 mm-thick sections were cut for retinas, and 14 mm-thick longitudinal sections were cut for optic nerves. For immunostaining, the sections were blocked in a staining buffer containing 3% bovine serum albumin, 5% normal goat serum, and 0.4% Triton X-100 in phosphate-buffered saline (PBS) for two hours. The sections were incubated with primary antibodies overnight at 4 °C and subsequently incubated with secondary antibodies at room temperature for two hours. For whole-mount retinas, fixed retinas were first radially cut into a petal shape (4 incisions) and blocked with PBS containing 3% bovine serum albumin, 5% normal goat serum, and 0.6% Triton X-100 for two hours at room temperature. Then, retinas were incubated with primary antibodies in a blocking buffer for two days at 4 °C. Next, retinas were incubated with secondary antibodies in a blocking buffer for two hours at room temperature. Images were captured using a confocal laser microscopy (TCS SP8, Leica, Germany).

For the immunostaining of retina explants, we used a previously published protocol [26, 42]. First, retinal explants were fixed in 3% PFA/3% sucrose for 15 min, then blocked with PBS containing 3% bovine serum albumin, 5% normal goat serum, and 0.1% Triton X-100 for 10 min at room temperature. Next, the explants were incubated with a primary antibody (anti-beta III tubulin, Tuj1) for two hours. Finally, the explants were washed three times for 10 min each with PBS and incubated with secondary antibodies and phalloidin (Invitrogen, R415) for one hour.

Primary antibodies used: Guinea pig anti-RBPMS (1:200, MilliporeSigma, ABN1376), Mouse anti-beta III tubulin (Tuj1, 1:300, Abcam, ab78078), Rabbit anti-Porf-2 (1:200, Abcam, ab93780), Rabbit anti-pGSK3β (Ser9) (1:200, Cell Signaling Technology, 5558), Rabbit anti-phospho-S6 ribosomal protein (Ser235/236) (1:200, Cell Signaling Technology, 4858). Secondary antibodies used: Goat anti-Mouse IgG (H + L) Alexa Fluor 488 (1:500, Thermo Fisher Scientific, A11001), Goat anti-Rabbit IgG (H + L) Alexa Fluor 555 (1:500, Thermo Fisher Scientific, A21428), Goat anti-Mouse IgG (H + L) Alexa Fluor 633 (1:500, Thermo Fisher Scientific, A21050), Goat anti-Guinea pig 488 (1:500, Abcam, ab150185), and Goat anti-Guinea pig 647 (1:500, Abcam, ab150187).

### Western blot analysis of mTORC1, GSK3β and STAT3 pathway-related proteins

Total protein was extracted from retinal tissues using the RIPA buffer (Epizyme, PC102) supplemented with protease inhibitor and phosphatase inhibitor cocktail. The extracted proteins were then separated by 10% gradient SDS-PAGE gel electrophoresis and transferred onto polyvinylidene fluoride membranes. Subsequently, the membranes were blocked for 2 h in TBST (150 mM NaCl, 10 mM Tris, 0.1% Tween 20, pH 7.6) containing 10% BSA. Primary antibodies were diluted in blocking buffer and incubated with the membranes overnight at 4 °C. After washing the membranes with TBST three times, they were incubated with HRP-conjugated secondary antibodies for 1 h at room temperature. Following another round of washing with TBST three times, the blots were exposed to an enhanced chemiluminescence substrate. Quantification of the bands was performed by analyzing the relative density of the exposed film using Image J software. The primary antibodies used for Western blotting were as follows: anti-phospho-STAT3 antibody (1:1000, Cell Signaling Technology, 9145), anti-STAT3 antibody (1:1000, Cell Signaling Technology, 9139), anti-phospho-GSK3β antibody (1:1000, Cell Signaling Technology, 5558), anti-GSK3β antibody (1:1000, Cell Signaling Technology, 12456), anti-phospho-S6 antibody (1:1000, Cell Signaling Technology, 5364 s), anti-S6 antibody (1:1000, Cell Signaling Technology, 2217 s), anti-phospho-4EBP1 antibody (1:1000, Cell Signaling Technology, 2855), anti-4EBP1 antibody (1:1000, Cell Signaling Technology, 9644), and anti-GAPDH antibody (1:1000, Cell Signaling Technology, 51332).

### Quantification of fluorescence intensity, RGC survival rate, RGC axon regeneration, retinal explant growth, and axon growth cone formation

Fluorescence images were taken in the same configuration for all retinas, and the confocal settings were kept constant for all scans. Fluorescence intensity was analyzed using ImageJ software. In all sections, RGCs were circled according to their morphology based on RBPMS or Tuj1 staining. To quantify the fluorescence intensity of Porf-2, pS6, or pGSK3β in all RGCs, five mice from each group were used, and at least eight non-adjacent retinal sections acquired with identical imaging settings were analyzed for each mouse.

To quantify RGC survival rate, retinal sections and whole-mounts were immunostained with an anti-RBPMS or anti-Tuj1 antibody. For the retinal sections, at least eight non-adjacent retinal sections were analyzed for each mouse. For retinal whole-mounts, the entire retina was divided into four quadrants centered on the optic papilla, and a consistent number of fields (six to nine) were selected in the center, middle, and periphery of each quadrant. Only RGCs in the ganglion cell layer were counted.

Regenerating RGC axons in injured optic nerves distal to the crush site were quantified as described previously [2, 7]. The number of CTB-labeled axons was estimated by counting the number of CTB-labeled fibers extending different distances from the end of the crush site in four sections per optic nerve. The cross-sectional width of the nerve was measured at the counting point to calculate the number of axons per millimeter of nerve width. The number of axons per millimeter was averaged over all sections. Σa_d_, the total number of axons extending distance d in a nerve with a radius of r, was estimated by summing over all the sections of a thickness t (14 µm): Σa_d_ = πr^2^ x [average axons/mm]/t.

Quantification of retinal explant growth and axon growth cone formation was performed as described previously [26, 42]. Anti-Tuj1 and Phalloidin were used to mark the morphology of axons and the growth cones in retinal explants, respectively. The axon outgrowth from retinal explants and growth cone area were quantified using ImageJ software.

### Analysis of RGC transduction rate

Analysis of the RGC transduction rate was conducted based on previous studies [2, 24]. Uninjured left retinas were taken from transcardially perfused mice two weeks after intravitreal AAV2-EGFP vector injection. The retinas were stained with a guinea pig anti-RBPMS antibody (1:200, MilliporeSigma, ABN1376) following the steps described above. For each mouse, the RGC transduction rate was calculated by dividing the total number of EGFP^+^ and RBPMS^+^ co-labeled cells by the total number of RBPMS^+^ cells. Only cells in the GCL were counted.

### Drug handling and administration

The Rac1 inhibitor, NSC23766 (Selleckchem, S8031), was administered as described previously [37, 38]. For in vivo studies, 4.0 mg/kg NSC23766 or the vehicle control was administered intraperitoneally once every two days following the AAV2-shPorf-2 injection. For in vitro experiments, 30 μM NSC23766 or the vehicle was added to the retinal explant medium, and the medium was changed once every two days. The detailed culture methods for retinal explants are described above.

### Rac1 activation assay and western blot analysis

Rac1 activity was assessed by calculating Rac1-GTP/Total Rac1 using the Rac1 Activation Assay Kit (Cytoskeleton, BK035-S) according to the manufacturer’s instructions. First, total protein was extracted from retinal tissues using the lysis buffer with the protease inhibitor and phosphatase inhibitor. 300–800 µg total protein was incubated with 10 µg PAK-PBD beads to pull down activated Rac1 proteins. After incubating at 4 °C on a rotator or rocker for 1 h, the PAK-PBD beads were pelleted by centrifugation at 5000 × *g* at 4 °C for 1 min. Next, 90% of the supernatant was removed very carefully, and the PAK-PBD beads were washed three times with a cold wash buffer. Then, 10–20 µl of 2x laemmli sample buffer was added to each tube, and the beads were thoroughly resuspended. Finally, the bead samples were boiled for 2 min prior to sodium dodecyl sulfate-polyacrylamide gel electrophoresis (SDS-PAGE) and Western blot (WB) analyses.

The extracted proteins were separated using SDS-PAGE and transferred onto polyvinylidene fluoride membranes. Then, membranes were blocked for 2 h in TBST (150 mM NaCl, 10 mM Tris, 0.1% Tween 20, and pH 7.6) containing 10% BSA. Primary antibodies were diluted in blocking buffer and incubated with the membrane overnight at 4 °C. After washing with TBST three times, the blots were incubated with horseradish peroxidase-conjugated secondary antibodies for 1 h at room temperature. After washing with TBST three times, the blots were exposed to an enhanced chemiluminescence substrate. Quantification was performed by analyzing the relative density of the exposed film using Image J. The following primary antibodies were used for WB: anti-Rac1 monoclonal antibody (1:1000, cytoskeleton, Cat # ARC03) and anti-GAPDH (1:1000, arigo, ARG10112).

### SD-OCT examination

SD-OCT (Micron IV; Phoenix Research Laboratories, Pleasanton, CA, USA) was used to measure the changes in GCC thickness before and three weeks after ONC injury, as previously described [2, 43]. Briefly, radial volume scans (centered on the optic disc, with a diameter of 1.2 mm) were performed, and each volume consisted of 100 B-scans with 1000 A-scans per B-scan. Four images (scans 1, 26, 51, and 76 at 0°, 45°, 90°, and 135° in en face images) were analyzed using InSight software (version 1.1.5207, Phoenix Research Laboratories) and used for retinal thickness measurements. For each selected image, a vertical caliper was placed on each side of the optic nerve head (ONH) 500 µm away from the center of the ONH. The caliper was used to measure the thickness of the GCC, which consisted of the three innermost retinal layers: the NFL, GCL, and IPL. The GCC thickness of each retina was taken as the average of a total of eight measurements. The image analysis was performed in a blinded manner.

### pSTR-ERG examination

pSTR-ERG was performed to assess functional changes in RGCs before and three weeks after ONC injury, as previously reported [44, 45]. Mice were dark-adapted for 12 h and prepared for pSTR-ERG recording under dim red light. After the mice were anesthetized by intraperitoneal injection of sodium pentobarbital (100 mg/kg), their pupils were dilated with 0.5% tropicamide and 0.5% phenylephrine hydrochloride. The recording electrode attached to a contact lens was placed on the center of the cornea. A 2.5% hypromellose ophthalmic solution was applied to maintain hydration and conductivity between the cornea and the recording electrodes. The reference electrode was placed in the middle of the lower eyelid, and the ground electrode was placed near the tail of the mouse. Recordings were generated using the Espion Visual Electrophysiology System (Espion E3, Diagnosys, Diagnosys UK Ltd, UK). The ERG was recorded under dark adaptation with increasing stimulus intensity from 3 × 10^−5^cd.sm^−2^ to 0.03 cd.sm^−2^. The response to approximately 30 flashes was recorded at each stimulus intensity, with an interstimulus interval of 2 s, and then averaged to determine the final value of pSTR. The amplitude of pSTR was measured from the baseline to the peak of the first positive wave.

### PLR examination

PLR was performed to evaluate the functional responses of the retina and the optic nerve before and eight weeks after ONC injury, as previously reported [46, 47]. Before the experiment, animals were dark-adapted for at least 12 h. Under dim red illumination, each mouse was placed in a head-and-body restrainer device with a metal bar (implanted into the skull two days before the recording) fixed to the device and PLRs were recorded using a pupillometer (A2000; Neuroptics Inc., Irvine, CA, USA) in combination with Bandicam software (Bandicam Company, Seoul, South Korea). After 60 s of dark adaptation, PLR was recorded at a light intensity of 1 W/m^2,^ and the stimulus was presented for 20 sec. Pupil area was then quantified manually using ImageJ software. The percent change in pupil area was calculated by recording the pupil area in the dark, subtracting the pupil area in the light, dividing the difference by the pupil area in the dark, and multiplying by 100. To facilitate a better understanding of the variations in pupil constriction among the different groups, we normalized the results to the pupil constriction in the intact group. This normalization enables a comparison of relative changes in pupil size across the various experimental conditions. The mean percent constriction of each pupil was utilized for statistical analyses.

### Statistical analysis

GraphPad Prism software was used for statistical analysis. All statistical details of each experiment are depicted in the figure legends. The data shown in the graphs are presented as the mean ± SEM. Two-tailed unpaired *t*-tests or Mann–Whitney test were used for comparisons between the two groups. Comparisons between two groups at multiple time points were analyzed via two-way analysis of variance (ANOVA) followed by Bonferroni’s multiple-comparisons test. One-way ANOVA with Bonferroni’s multiple-comparisons test was used to compare multiple groups. A *P*-value % 0.05 was considered statistically significant.

## Supplementary information

Supplementary figure legends

Supplementary Fig1

Supplementary Fig2

Supplementary Fig3

Supplementary Fig4

Supplementary Fig5

Supplementary Fig6

Reproducibility checklist

Original Data File

## Data Availability

All data generated or analyzed during this study are included in this published article and supplementary information files.

## References

[1] Guo X, Zhou J, Starr C, Mohns EJ, Li Y, Chen EP (2021). Preservation of vision after CaMKII-mediated protection of retinal ganglion cells. Cell..

[2] Chen D, Sun YY, Zhou LY, Yang S, Hong FY, Liu XD (2022). Maf1 regulates axonal regeneration of retinal ganglion cells after injury. Exp Neurol.

[3] Jacobi A, Tran NM, Yan W, Benhar I, Tian F, Schaffer R (2022). Overlapping transcriptional programs promote survival and axonal regeneration of injured retinal ganglion cells. Neuron..

[4] Lu Y, Brommer B, Tian X (2020). Reprogramming to recover youthful epigenetic information and restore vision. Nature..

[5] Benowitz LI, He Z, Goldberg JL (2017). Reaching the brain: Advances in optic nerve regeneration. Exp Neurol.

[6] Park KK, Liu K, Hu Y, Smith PD, Wang C, Cai B (2008). Promoting axon regeneration in the adult CNS by modulation of the PTEN/mTOR pathway. Science..

[7] Smith PD, Sun F, Park KK, Cai B, Wang C, Kuwako K (2009). SOCS3 deletion promotes optic nerve regeneration in vivo. Neuron..

[8] Guo X, Snider WD, Chen B (2016). GSK3beta regulates AKT-induced central nervous system axon regeneration via an eIF2Bepsilon-dependent, mTORC1-independent pathway. eLife..

[9] Sergeeva EG, Rosenberg PA, Benowitz LI (2021). Non-Cell-Autonomous Regulation of Optic Nerve Regeneration by Amacrine Cells. Front Cell Neurosci.

[10] Au NPB, Chand R, Kumar G, Asthana P, Tam WY, Tang KM (2022). A small molecule M1 promotes optic nerve regeneration to restore target-specific neural activity and visual function. Proc Natl Acad Sci USA.

[11] de Lima S, Koriyama Y, Kurimoto T, Oliveira JT, Yin Y, Li Y (2012). Full-length axon regeneration in the adult mouse optic nerve and partial recovery of simple visual behaviors. Proc Natl Acad Sci USA.

[12] Nowak FV (2018). Porf-2 = Arhgap39 = Vilse: A Pivotal Role in Neurodevelopment, Learning and Memory. eNeuro.

[13] Nowak FV (1990). Cloning of two hypothalamic cDNAs encoding tissue-specific transcripts in the preoptic area and testis. Mol Endocrinol.

[14] Ma S, Nowak FV (2011). The RhoGAP domain-containing protein, Porf-2, inhibits proliferation and enhances apoptosis in neural stem cells. Mol Cell Neurosci.

[15] Huang GH, Yang XT, Chen K, Xing J, Guo L, Zhu L (2016). Porf-2 Inhibits Neural Stem Cell Proliferation Through Wnt/beta-Catenin Pathway by Its GAP Domain. Front Cell Neurosci.

[16] Yang XT, Huang GH, Li HJ, Sun ZL, Xu NJ, Feng DF (2017). Rac1 Guides Porf-2 to Wnt Pathway to Mediate Neural Stem Cell Proliferation. Front Mol Neurosci.

[17] Huang GH, Sun ZL, Li HJ, Feng DF (2017). Rho GTPase-activating proteins: Regulators of Rho GTPase activity in neuronal development and CNS diseases. Mol Cell Neurosci.

[18] Lim J, Ritt DA, Zhou M, Morrison DK (2014). The CNK2 scaffold interacts with vilse and modulates Rac cycling during spine morphogenesis in hippocampal neurons. Curr Biol.

[19] Lundström A, Gallio M, Englund C, Steneberg P, Hemphälä J, Aspenström P (2004). Vilse, a conserved Rac/Cdc42 GAP mediating Robo repulsion in tracheal cells and axons. Genes Dev.

[20] Hu H, Li M, Labrador JP, McEwen J, Lai EC, Goodman CS (2005). Cross GTPase-activating protein (CrossGAP)/Vilse links the Roundabout receptor to Rac to regulate midline repulsion. Proc Natl Acad Sci USA.

[21] Huang GH, Guo L, Zhu L, Liu XD, Sun ZL, Li HJ (2018). Neuronal GAP-Porf-2 transduces EphB1 signaling to brake axon growth. Cell Mol Life Sci.

[22] Geoffroy CG, Hilton BJ, Tetzlaff W, Zheng B (2016). Evidence for an Age-Dependent Decline in Axon Regeneration in the Adult Mammalian Central Nervous System. Cell Rep.

[23] Yao K, Qiu S, Wang YV, Park SJH, Mohns EJ, Mehta B (2018). Restoration of vision after de novo genesis of rod photoreceptors in mammalian retinas. Nature..

[24] Wang XW, Yang SG, Zhang C, Hu MW, Qian J, Ma JJ (2020). Knocking Out Non-muscle Myosin II in Retinal Ganglion Cells Promotes Long-Distance Optic Nerve Regeneration. Cell Rep.

[25] Tedeschi A, Dupraz S, Curcio M, Laskowski CJ, Schaffran B, Flynn KC (2019). ADF/Cofilin-Mediated Actin Turnover Promotes Axon Regeneration in the Adult CNS. Neuron..

[26] Nawabi H, Belin S, Cartoni R, Williams PR, Wang C, Latremoliere A (2015). Doublecortin-Like Kinases Promote Neuronal Survival and Induce Growth Cone Reformation via Distinct Mechanisms. Neuron..

[27] Pita-Thomas W, Mahar M, Joshi A, Gan D, Cavalli V (2019). HDAC5 promotes optic nerve regeneration by activating the mTOR pathway. Exp Neurol.

[28] Leibinger M, Hilla AM (2019). GSK3-CRMP2 signaling mediates axonal regeneration induced by Pten knockout. Mol Neurobiol.

[29] Kumar V, Zhang MX, Swank MW, Kunz J, Wu GY (2005). Regulation of dendritic morphogenesis by Ras-PI3K-Akt-mTOR and Ras-MAPK signaling pathways. J Neurosci.

[30] Chen K, Zhu L, Guo L, Pan YB, Feng DF (2020). Maf1 regulates dendritic morphogenesis and influences learning and memory. Cell Death Dis.

[31] Zhang Y, Williams PR, Jacobi A, Wang C, Goel A, Hirano AA (2019). Elevating Growth Factor Responsiveness and Axon Regeneration by Modulating Presynaptic Inputs. Nat Neurosci.

[32] Li XY, Huang GH, Liu QK, Yang XT, Wang K, Luo WZ (2020). Porf-2 Inhibits Tumor Cell Migration Through the MMP-2/9 Signaling Pathway in Neuroblastoma and Glioma. Front Oncol.

[33] Belin S, Norsworthy M, He Z (2014). Independent control of aging and axon regeneration. Cell Metab.

[34] Liu K, Lu Y, Lee JK, Samara R, Willenberg R, Sears-Kraxberger I (2010). PTEN deletion enhances the regenerative ability of adult corticospinal neurons. Nat Neurosci.

[35] Lorenzetto E, Ettorre M, Pontelli V, Bolomini-Vittori M, Bolognin S, Zorzan S (2013). Rac1 selective activation improves retina ganglion cell survival and regeneration. PLoS One.

[36] Matsukawa T, Morita K, Omizu S, Kato S, Koriyama Y (2018). Mechanisms of RhoA inactivation and CDC42 and Rac1 activation during zebrafish optic nerve regeneration. Neurochem Int.

[37] Bu F, Munshi Y, Furr JW, Min JW, Qi L, Patrizz A (2021). Activation of neuronal Ras-related C3 botulinum toxin substrate 1 (Rac1) improves post-stroke recovery and axonal plasticity in mice. J Neurochem.

[38] Liu L, Yuan H, Yi Y, Koellhoffer EC, Munshi Y, Bu F (2018). Ras-Related C3 Botulinum Toxin Substrate 1 Promotes Axonal Regeneration after Stroke in Mice. Transl Stroke Res.

[39] McCown TJ (2011). Adeno-Associated Virus (AAV) Vectors in the CNS. Curr Gene Ther.

[40] Ojala DS, Amara DP, Schaffer DV (2015). Adeno-associated virus vectors and neurological gene therapy. Neuroscientist..

[41] Alarautalahti V, Ragauskas S, Hakkarainen JJ, Uusitalo-Järvinen H, Uusitalo H, Hyttinen J (2019). Viability of Mouse Retinal Explant Cultures Assessed by Preservation of Functionality and Morphology. Invest Ophthalmol Vis Sci.

[42] Schaeffer J, Delpech C, Albert F, Belin S, Nawabi H (2020). Adult Mouse Retina Explants: From ex vivo to in vivo Model of Central Nervous System Injuries. Front Mol Neurosci.

[43] Li HJ, Sun ZL, Pan YB, Sun YY, Xu MH, Feng DF (2019). Inhibition of miRNA-21 promotes retinal ganglion cell survival and visual function by modulating Müller cell gliosis after optic nerve crush. Exp Cell Res.

[44] Liu Y, McDowell CM, Zhang Z, Tebow HE, Wordinger RJ, Clark AF (2014). Monitoring retinal morphologic and functional changes in mice following optic nerve crush. Invest Ophthalmol Vis Sci.

[45] Yukita M, Machida S, Nishiguchi KM, Tsuda S, Yokoyama Y, Yasuda M (2015). Molecular, anatomical and functional changes in the retinal ganglion cells after optic nerve crush in mice. Doc Ophthalmol.

[46] Zuo L, Khan RS, Lee V, Dine K, Wu W, Shindler KS (2013). SIRT1 promotes RGC survival and delays loss of function following optic nerve crush. Invest Ophthalmol Vis Sci.

[47] Zhou W, Wang LQ, Shao YQ, Han X, Yu CX, Yuan F (2021). Orexin-A Intensifies Mouse Pupillary Light Response by Modulating Intrinsically Photosensitive Retinal Ganglion Cells. J Neurosci.

