# Supplementary material for: Knockdown of Porf-2 restores visual function after optic nerve crush injury
Source: Cell Death Dis. 2023 Aug 28;14(8):570. doi: 10.1038/s41419-023-06087-2 (PMC10462692; doi:10.1038/s41419-023-06087-2)
Supplement: Supplementary file 1 — Supplementary figure legends [file 41419_2023_6087_MOESM1_ESM.docx]

**Supplementary figure legends**

**Supplementary Fig.1** **Alteration of Porf-2 expression in RGCs before and 3 days after optic nerve crush (ONC). A, B** Representative confocal images showing the expression level of Porf-2 (red) in RBPMS^+^ (magenta) RGCs in the intact retina and at the third-day post-crush (3 dpc) by RNA-Scope (**A**) and immunofluorescence (**B**). Scale bar, 20 μm. **C** The rt-qPCR showing the relative expression of Porf-2 in retina before and 3 days after ONC (Mann Whitney test, p < 0.05, n = 4 independent experiments). **D, E** Quantification of the average fluorescence intensity of Porf-2 in RBPMS^+^ RGCs in (**A**) and (**B**) (Mann Whitney test, p < 0.05, n = 6 mice in each group of RNA-Scope, n = 8 mice in each group of immunofluorescence, six to eight fields were analyzed for each retina). Data are represented as mean ± SEM. *p < 0.05

**Supplementary Fig.2 High transduction rate of AAV2-shRNA-EGFP and downregulation of Porf-2 levels in RGCs two weeks after intravitreal injection of AAV2-shPorf-2.** **A** Representative confocal images of flat-mounted retinas showing AAV2-shRNA-EGFP positive (green) and anti-RBPMS positive (red) RGCs. Scale bar, 20 μm. **B** Representative confocal images of retinal sections showing the expression level of Porf-2 (red) in RBPMS^+^ RGCs (magenta). Scales bar, 20 μm. **C** Quantification of the transduction rate of AAV2-shRNA-EGFP in RGCs in (**A**). The average transduction rate was 91.00 ± 0.51% (n = 6 mice for each group; six to eight fields were analyzed for each retina). **D** Quantification of the average fluorescence intensity of Porf-2 in RBPMS^+^ RGCs in (**B**) (one-way ANOVA followed by Tukey’s multiple-comparisons test; n = 10 mice in each group, at least eight non-adjacent retinal sections were analyzed for each mouse). **E** The rt-qPCR showing the relative expression of Porf-2 in retina two weeks after injection of AAV2-shPorf-2 (one-way ANOVA followed by Bonferroni's multiple comparisons test, n = 4 independent experiments). Data are represented as mean ± SEM. *p < 0.05, **p < 0.01, ***p < 0.001.

**Supplementary Fig.3 Effects of injected and non-injected shCtrl on axonal regeneration, RGC survival, and explant growth after optic nerve injury. A** Representative confocal images of optic nerves from injected and non-injected shCtrl two weeks after ONC injury. Asterisks indicate the optic nerve crush site. Scale bar, 200 μm. **B** Quantification of optic nerve regeneration in (**A**) (two-way ANOVA followed by Bonferroni's multiple-comparisons test; n = 5 mice in each group). **C** Representative confocal images of retinal sections showing RBPMS+ RGCs (magenta) from injected and non-injected shCtrl two weeks after ONC injury. Scale bar, 20 μm. **D** Quantification of the RGC survival rate in (**C**) (Mann Whitney test; p > 0.05, n = 6 mice in each group, at least eight non-adjacent retinal sections were analyzed for each retina). **E** Representative microphotographs of retinal explants stained with anti-beta III tubulin (Tuj1) showing neurite growth in injected and non-injected shCtrl groups. Scale bar, 100 μm. **F** Quantification of neurites counted at different distances from the edges of the explants in (**E**) (two-way ANOVA followed by Bonferroni's multiple-comparisons test; n = 5 mice in each group). Data are presented as the mean ± SEM. ns, not significant.

**Supplementary Fig.4 Knockdown of Porf-2 promoted** **RGCs survival after ONC. A** Representative confocal images of flat-mounted retinas showing Porf-2-knockdown (AAV2-shRNA1 and AAV2-shRNA2) and control Tuj1^+^ RGCs (magenta) in the young mice two weeks after ONC. Scale bar, 20 μm. **B** Representative confocal images of optic nerves in Porf-2-knockdown RGCs at 8 weeks post-ONC injury. Asterisks indicate the optic nerve crush site. Scale bar, 500 μm. **C** Quantification of the RGC survival rate in (**A**) (one-way ANOVA followed by Bonferroni's multiple comparisons test, n = 6 mice in each group, six to eight fields were analyzed for each retina). Data are represented as mean ± SEM. ***p < 0.001, ****p < 0.0001.

**Supplementary Fig.5 Porf-2-knockdown-induced axon regeneration and RGCs survival was not mediated by mTORC1, GSK3β and STAT3 pathway. A, B** Representative confocal images of retinal sections showing pS6 (**A**) and GSK3β (**B**) expression level of Porf-2-knockdown and control RGCs in intact condition. Scale bar, 20 μm. **C** Representative western blot showing the changes of pS6, p4EBP1, pGSK3b and pSTAT3 after knockdown of Porf-2. **D, E** Quantification of the percentage of pS6^+^ RGCs in (**A**) and the average ﬂuorescence intensity of GSK3β in RBPMS^+^ RGCs in (**B**) (Mann Whitney test, p > 0.05, n = 6 mice for each group; at least eight non-adjacent retinal sections were analyzed for each mouse). **F-I** Quantification of the relative expression of pSTAT3/STAT3 (**F**), pGSK3b/GSK3b (**G**), pS6/S6 (**H**) and p4EBP1/4EBP1(**I**) in (**C**) (Mann Whitney test, p > 0.05, n = 3 independent experiments). Data are represented as mean ± SEM. ns, not significant.

**Supplementary Fig.6 Knockdown of Porf-2 improved the activity of Rac1. A** Representative western blot showing the alteration of Rac1 activity level of Porf-2-knockdown and control retina before and 3dpc after ONC. **B, C** Representative western blot showing the alteration of Rac1 activity level in mice treated with AAV2-shCtrl (**B**) or AAV2-shPorf-2 (**C**) after intraperitoneal injection of NSC23766 or vehicle, respectively. **D** Quantification of the relative Rac1 activity in (**A**) (two-way ANOVA followed by Bonferroni's multiple-comparisons test, P < 0.001; n = 5 independent experiments). **E, F** Quantification of the relative Rac1 activity in (**B**) and (**C**) (Mann Whitney test, p < 0.01, n = 5 independent experiments). Data are represented as mean ± SEM. **p < 0.01, ***p < 0.001.
