# Supplementary figures and images for: Knockdown of Porf-2 restores visual function after optic nerve crush injury
Source: Cell Death Dis. 2023 Aug 28;14(8):570. doi: 10.1038/s41419-023-06087-2 (PMC10462692; doi:10.1038/s41419-023-06087-2)

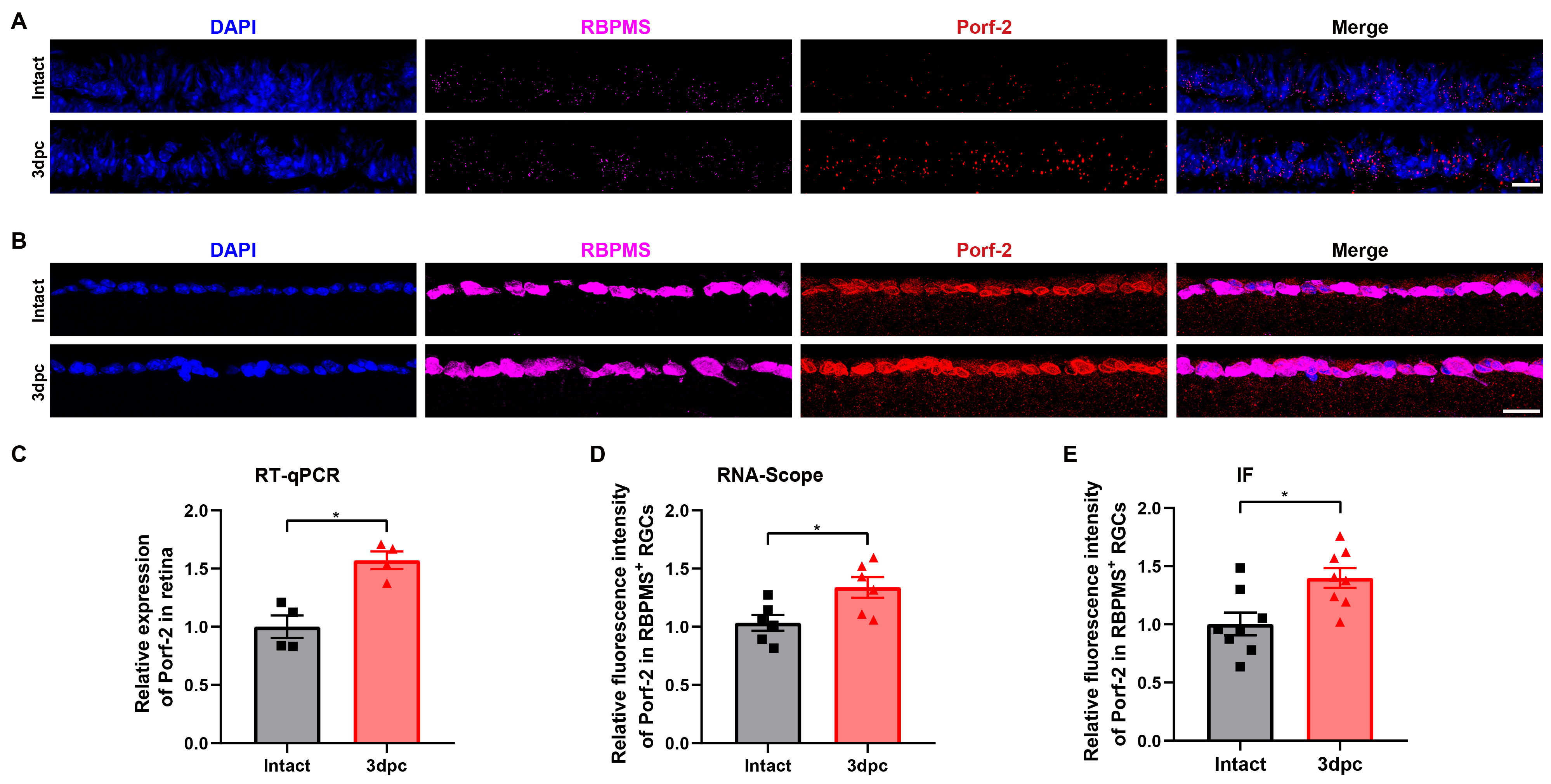

Supplement: Supplementary file 2 — Supplementary Fig1 [file 41419_2023_6087_MOESM2_ESM.tif]

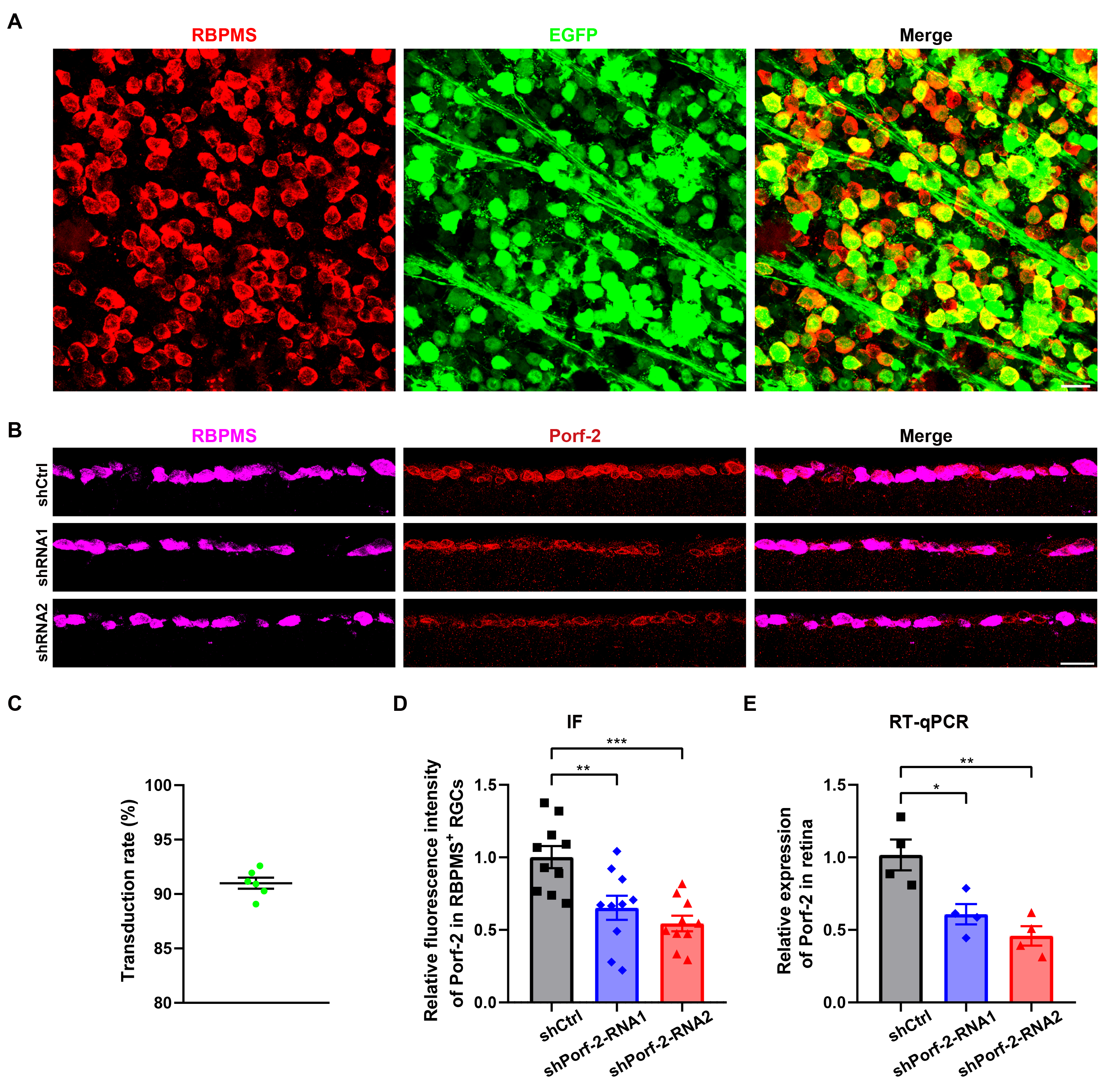

Supplement: Supplementary file 3 — Supplementary Fig2 [file 41419_2023_6087_MOESM3_ESM.tif]

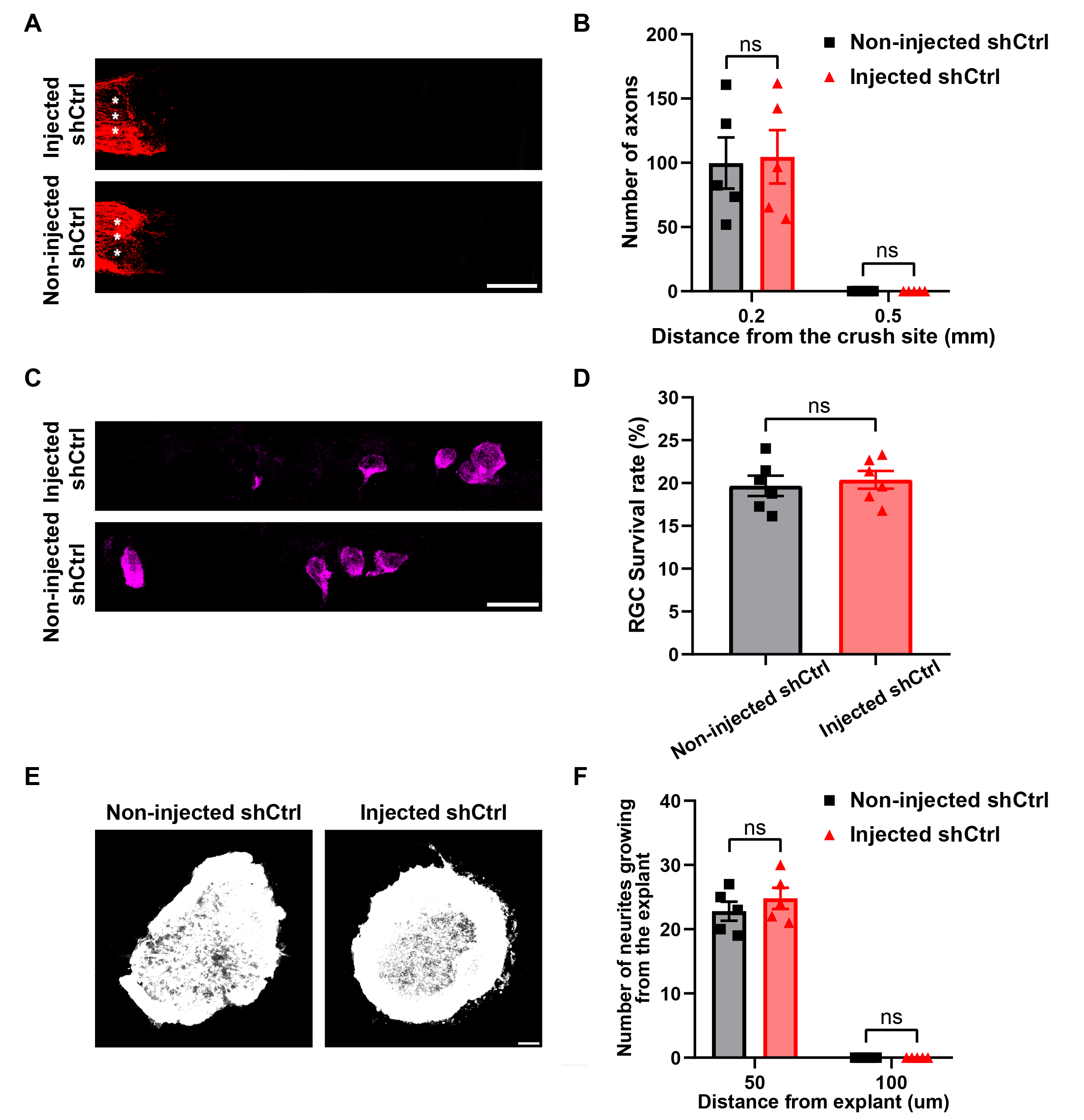

Supplement: Supplementary file 4 — Supplementary Fig3 [file 41419_2023_6087_MOESM4_ESM.tif]

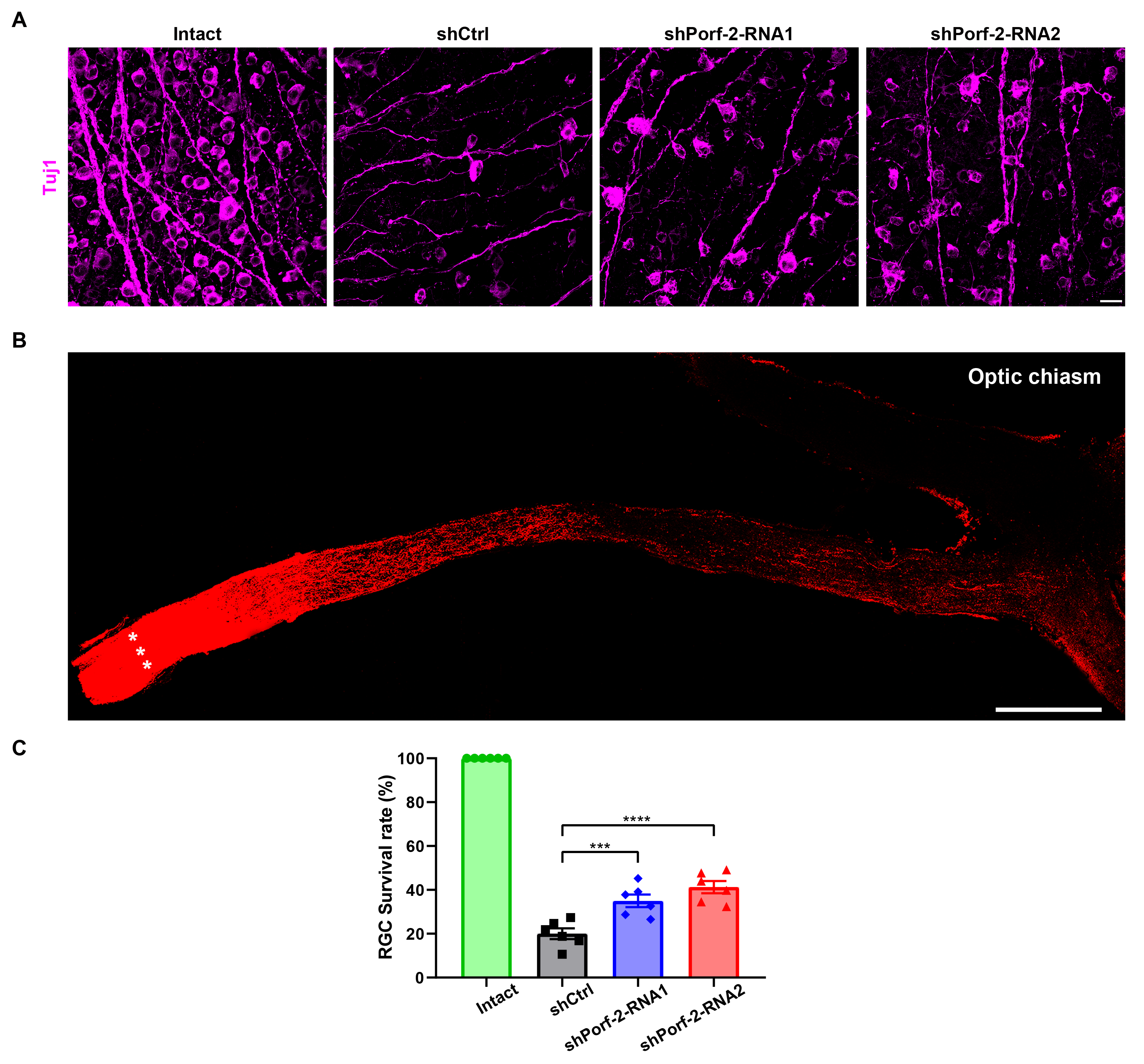

Supplement: Supplementary file 5 — Supplementary Fig4 [file 41419_2023_6087_MOESM5_ESM.tif]

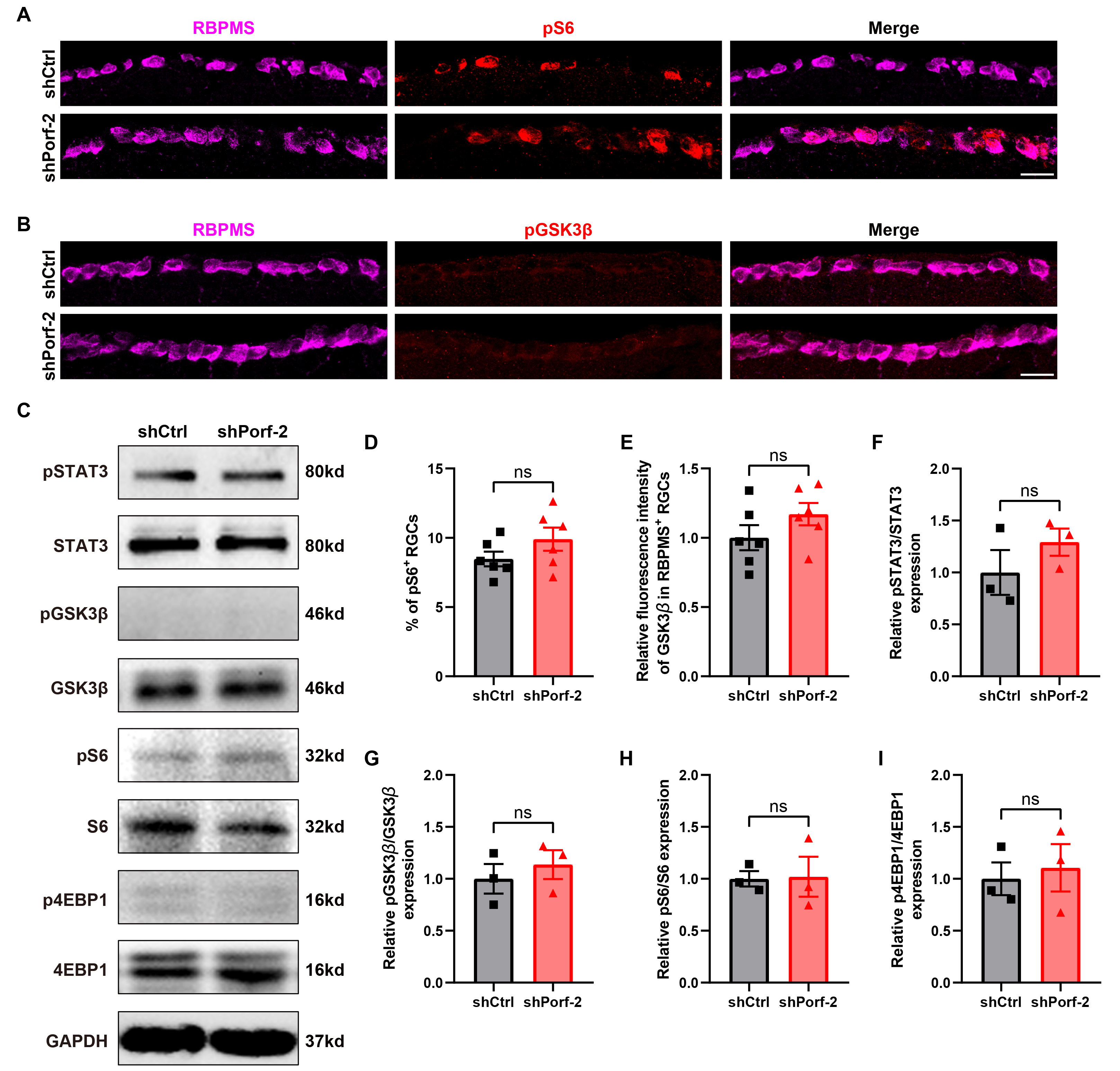

Supplement: Supplementary file 6 — Supplementary Fig5 [file 41419_2023_6087_MOESM6_ESM.tif]

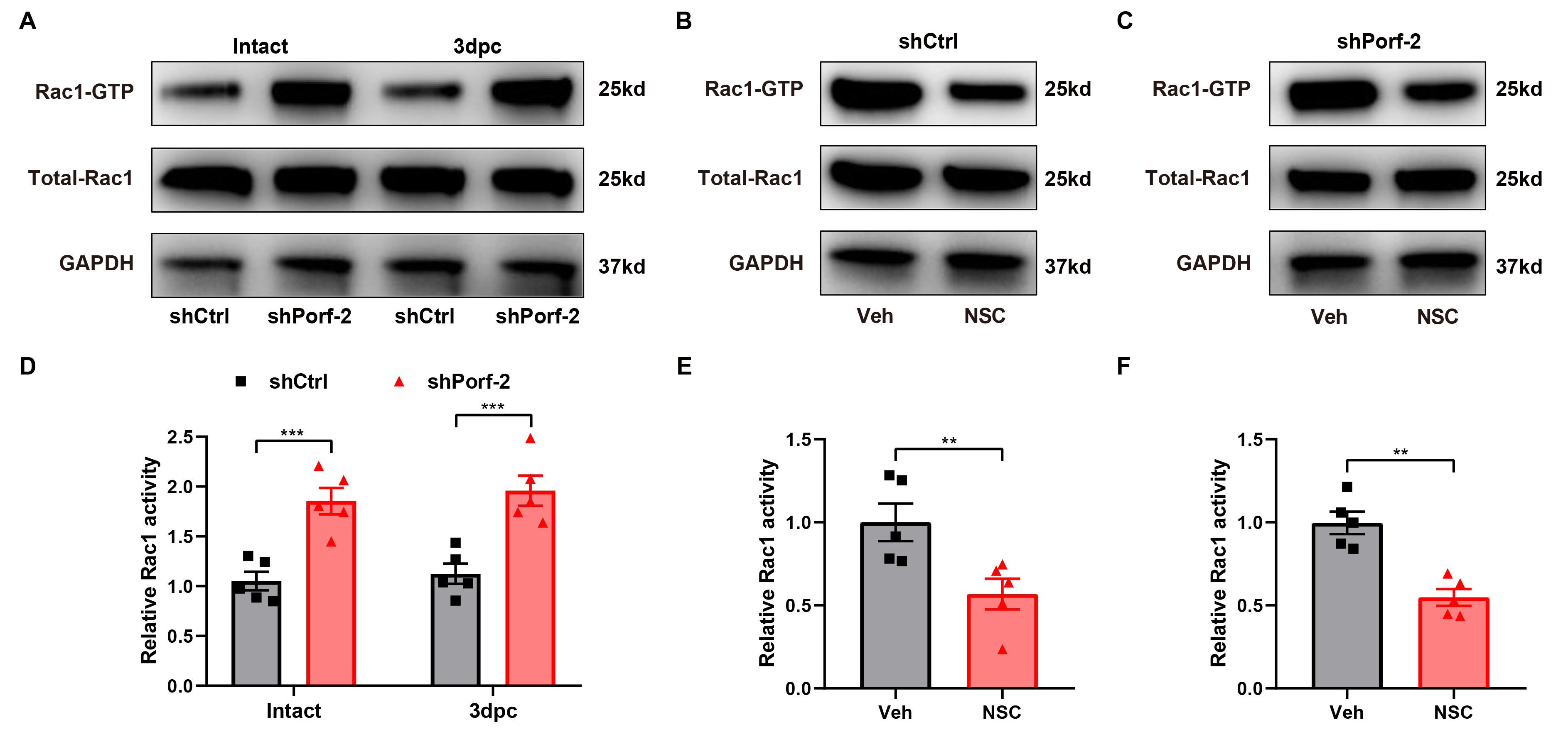

Supplement: Supplementary file 7 — Supplementary Fig6 [file 41419_2023_6087_MOESM7_ESM.tif]
