# Supplementary material for: Knockdown of Porf-2 restores visual function after optic nerve crush injury
Source: Cell Death Dis. 2023 Aug 28;14(8):570. doi: 10.1038/s41419-023-06087-2 (PMC10462692; doi:10.1038/s41419-023-06087-2)
Supplement: Supplementary file 9 — Original Data File [file 41419_2023_6087_MOESM9_ESM.docx]

**Original western blots:**


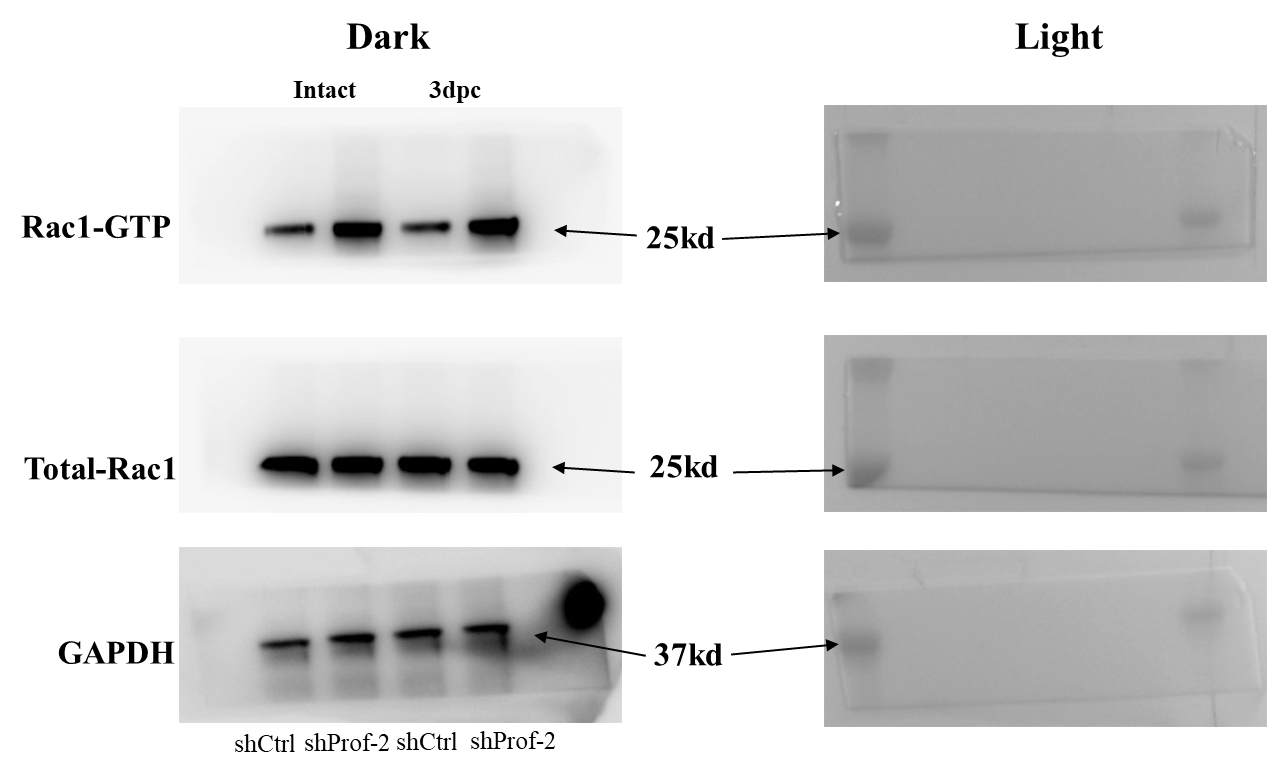


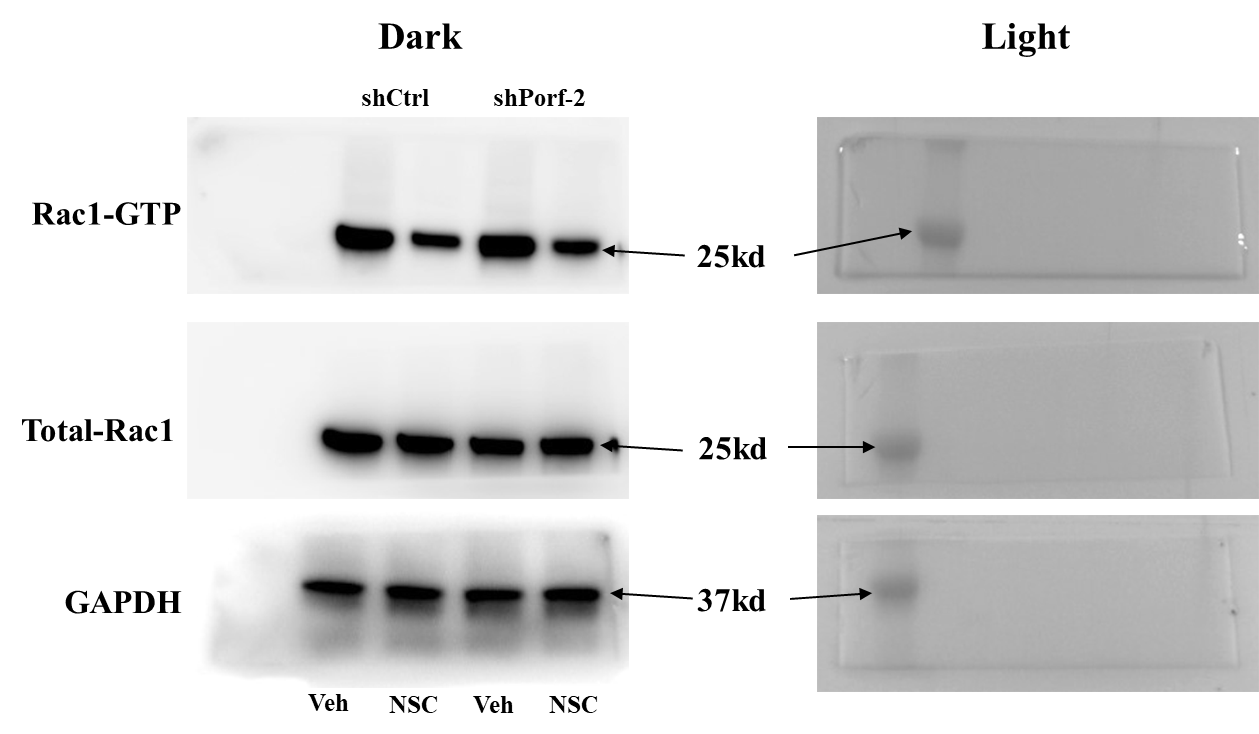


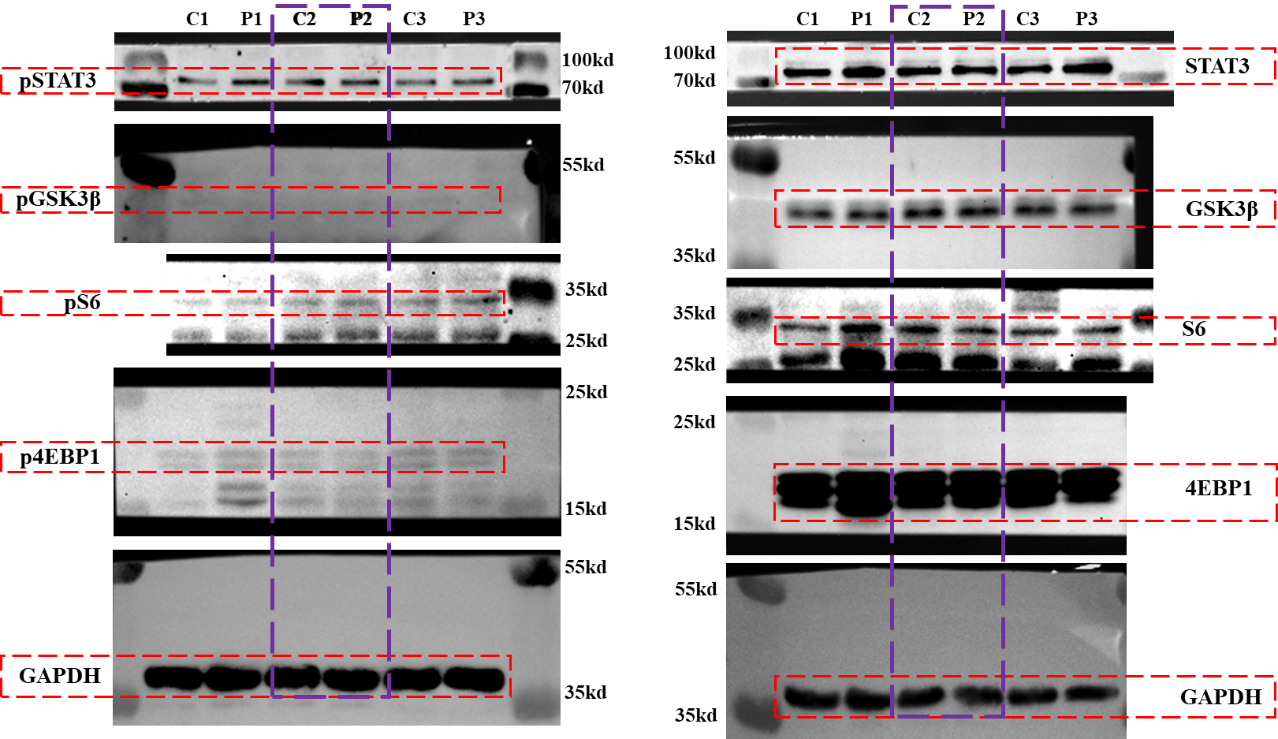


n = 3 independent experiments;

C: shCtrl; P: shPorf-2;

C2 and P2 are displayed in the revised manuscript as representative WB strips.
